# Functional *Pdgfra* fibroblast heterogeneity in normal and fibrotic mouse lung

**DOI:** 10.1172/jci.insight.164380

**Published:** 2023-11-22

**Authors:** Carol S. Trempus, Brian N. Papas, Maria I. Sifre, Carl D. Bortner, Erica Scappini, Charles J. Tucker, Xin Xu, Katina L. Johnson, Leesa J. Deterding, Jason G. Williams, Dylan J. Johnson, Jian-Liang Li, Deloris Sutton, Charan Ganta, Debabrata Mahapatra, Muhammad Arif, Abhishek Basu, Lenny Pommerolle, Resat Cinar, Anne K. Perl, Stavros Garantziotis

**Affiliations:** 1 Immunity, Inflammation, and Disease Laboratory,; 2 Biostatistics & Computational Biology Branch,; 3 Signal Transduction Laboratory, and; 4 Epigenetics & Stem Cell Biology Laboratory, National Institute of Environmental Health Sciences, NIH, Research Triangle Park, North Carolina, USA.; 5 Comparative & Molecular Pathogenesis Branch, National Institute of Environmental Health Sciences, Division of Translational Toxicology, Research Triangle Park, North Carolina, USA.; 6 Inotiv, Research Triangle Park, North Carolina, USA.; 7 Section on Fibrotic Disorders, and; 8 Laboratory of Cardiovascular Physiology and Tissue Injury, National Institute on Alcohol Abuse and Alcoholism, NIH, Rockville, Maryland, USA.; 9 Division of Pulmonary Biology, Department of Pediatrics, University of Cincinnati College of Medicine, Cincinnati Children’s Hospital Medical Center, Cincinnati, Ohio, USA.

**Keywords:** Pulmonology, Fibrosis

## Abstract

Aberrant fibroblast function plays a key role in the pathogenesis of idiopathic pulmonary fibrosis, a devastating disease of unrelenting extracellular matrix deposition in response to lung injury. Platelet-derived growth factor α–positive (*Pdgfra*^+^) lipofibroblasts (LipoFBs) are essential for lung injury response and maintenance of a functional alveolar stem cell niche. Little is known about the effects of lung injury on LipoFB function. Here, we used single-cell RNA-Seq (scRNA-Seq) technology and *Pdgfra^GFP^* lineage tracing to generate a transcriptomic profile of *Pdgfra*^+^ fibroblasts in normal and injured mouse lungs 14 days after bleomycin exposure, generating 11 unique transcriptomic clusters that segregated according to treatment. While normal and injured LipoFBs shared a common gene signature, injured LipoFBs acquired fibrogenic pathway activity with an attenuation of lipogenic pathways. In a 3D organoid model, injured *Pdgfra*^+^ fibroblast–supported organoids were morphologically distinct from those cultured with normal fibroblasts, and scRNA-Seq analysis suggested distinct transcriptomic changes in alveolar epithelia supported by injured *Pdgfra*^+^ fibroblasts. In summary, while LipoFBs in injured lung have not migrated from their niche and retain their lipogenic identity, they acquire a potentially reversible fibrogenic profile, which may alter the kinetics of epithelial regeneration and potentially contribute to dysregulated repair, leading to fibrosis.

## Graphical abstract

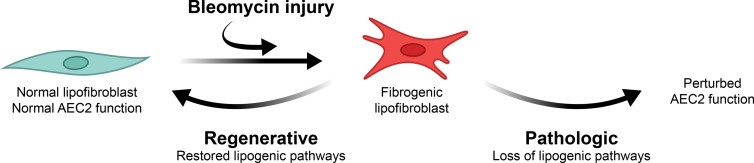

## Introduction

Idiopathic pulmonary fibrosis (IPF) is a devastating lung disease characterized by dysregulation of mesenchymal cells at sites of chronic epithelial injury, resulting in excessive extracellular matrix (ECM) deposition, aberrant remodeling, and loss of gas exchange regions of the lung, which often leads to respiratory failure and death 3–5 years from diagnosis (1–3).

Lung fibrosis results from a failure to maintain homeostasis in ECM turnover (4). The lungs are constantly exposed to the environment, and therefore subject to recurrent microscopic injury. Homeostasis requires maintenance of the normal structure of differentiated lung epithelium (i.e., regeneration of damaged alveoli) and orderly removal of provisional ECM. Lung fibroblasts (FBs) are a central mediator in these processes (5); hence, understanding their behavior and fate in lung injury is of paramount importance.

FBs expressing the gene encoding platelet-derived growth factor receptor α (*Pdgfra*^+^ FBs) are a versatile cell population with crucial roles in lung development and injury response and have therefore been called a “jack of all trades” cell population (6). For example, *Pdgfra*^+^ FBs are required for formation of secondary alveolar septa in mice, suggesting a central role in lung development (7, 8). Lipofibroblasts (LipoFBs, a subpopulation of *Pdgfra*^+^ FBs) are an indispensable constituent of the alveolar stem cell niche, where they are required for both alveolar homeostasis and epithelial regeneration after injury (9–12). LipoFBs can also give rise to myofibroblasts (MyoFBs) (13), contributing to both normal wound healing and to the pathological remodeling seen in fibrotic lungs. Because lung LipoFBs have essential roles in alveolar homeostasis, epithelial repair after injury, and fibrosis, it is imperative to understand the mechanisms determining the behavior of this population, and whether these mechanisms can be manipulated toward a less fibrotic, more homeostatic, phenotype.

In this study, we established transcriptomic single-cell RNA-Seq (scRNA-Seq) profiles for *Pdgfra*^+^ FBs in the naive state and after bleomycin-induced injury. We identified and characterized transcriptomic signatures defining normal and fibrotic LipoFBs, predicted key pathways regulating lipogenic and fibrogenic pathways in normal and fibrotic LipoFBs, and used 3D organoid culture to assess fibrotic LipoFB function in alveolar progenitor cell self-renewal and differentiation. Our data provide insights into fibrotic activation of LipoFBs after lung injury and identify possible targets for therapeutic intervention to ameliorate fibrosis.

## Results

### Identification of Pdgfra^+^ subpopulations in normal and fibrotic mouse lung.

**Figure 1:**
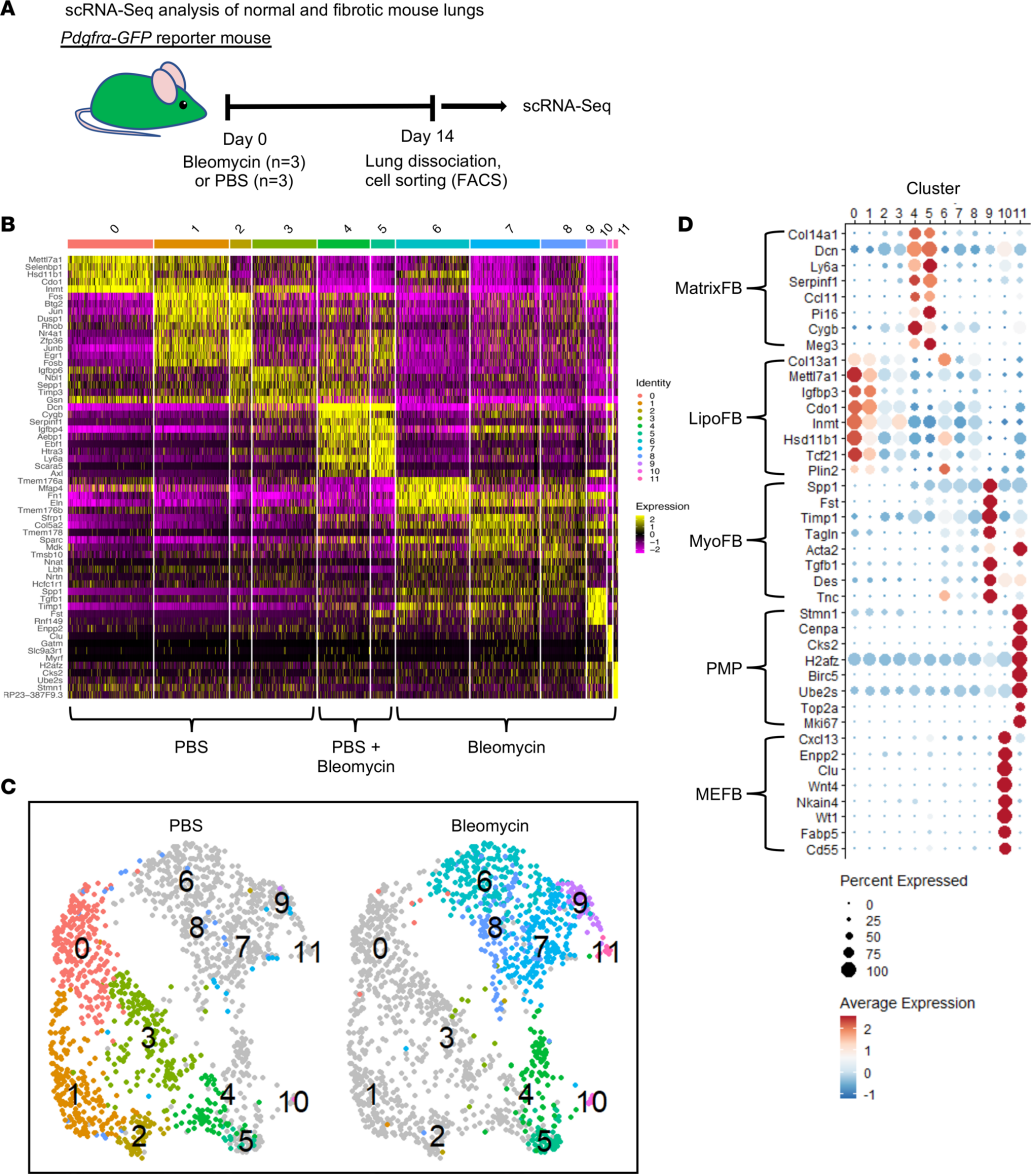
*Pdgfra*^+^ populations in normal and fibrotic mouse lung. (**A**) Diagram of experimental workflow. Data were generated from 3 PBS- and 3 bleomycin-treated mice, pooled for isolation of GFP^+^ cells. (**B**) Integrated heatmap of PBS- and bleomycin-derived scRNA-Seq analysis depicting 11 unique clusters based on individual cluster markers. (**C**) Uniform manifold approximation and projection (UMAP) representation of cluster distribution across treatment groups. (**D**) Dot plot representation of individual clusters to establish identification of cluster subtypes: matrix fibroblasts (MatrixFB), lipofibroblasts (LipoFB), myofibroblasts (MyoFB), proliferating mesenchymal progenitors (PMP), and mesothelial fibroblasts (MEFB).

Using the *Pdgfra^GFP^* reporter mouse, we undertook scRNA-Seq analysis and in silico modeling to investigate functional heterogeneity and response to injury within *Pdgfra*^+^ populations in normal and fibrotic lung. The experimental timeline and gating strategy for isolation and collection of live cells for scRNA-Seq are shown in Figure 1A and Supplemental Figure 1 (supplemental material available online with this article; https://doi.org/10.1172/jci.insight.164380DS1). Briefly, mice were dosed with bleomycin (2 U/kg) or PBS via oropharyngeal instillation, and lungs were collected on day 14 after exposure. Following enzymatic dissociation, GFP^+^ cells were isolated by FACS, and live cells were used for scRNA-Seq analysis. Localization of GFP*^+^* cells in normal and fibrotic lung was confirmed by immunofluorescence (Supplemental Figure 2). Masson’s trichrome staining of FFPE lungs from mice dosed with bleomycin or PBS confirmed collagen deposition and the development of a fibrotic response (Supplemental Figure 3). Quality control data are provided in Supplemental Table 1. GFP^+^ FBs from normal and fibrotic lungs formed 11 unique transcriptomic clusters (Figure 1B; marker lists are available in Supplemental Table 2). The clusters separated in uniform manifold approximation and projection (UMAP) space (Figure 1C) and segregated according to treatment. Clusters 0 through 3 derived predominantly from control lungs, clusters 4 and 5 contained cells from control and injured lungs, and clusters 6 through 11 contained cells from bleomycin-exposed lung (Supplemental Figure 4, A and B, cell proportions and number of cells, respectively).

*Pdgfra*^+^ FBs are a heterogeneous population (7, 14, 15), and known populations were identified using signature gene markers for matrix FBs (MatrixFBs), LipoFBs, MyoFBs, proliferating mesenchymal progenitors (PMPs), and mesothelial FBs (MEFBs) (Figure 1D). Clusters 4 and 5, which contain cells from control and injured lungs, represented MatrixFBs, also known as adventitial FBs or alveolar FB 2 (expressing *Col14a1*, *Dcn*, and *Ly6a*). LipoFB markers were predominantly found in normal lung clusters 0–3 and their expression was reduced in injured lung clusters 6–8, suggesting a significant attenuation of the transcriptomic signature of LipoFBs upon bleomycin-induced injury. Cluster 9 contains MyoFBs (*Spp1*, *Tagln*, and *Tgfb1*), cluster 10 MEFBs (*Clu*, *Wnt4*, and *Wt1*), and cluster 11 PMPs (*Mki67*, *Top2a*, and *Ube2s*) (14, 16, 17). Further analysis focused on the unique LipoFB clusters derived from both PBS (clusters 0–3) and bleomycin (clusters 6–8), based on canonical markers such as *Plin2* (*Adrp*) (18) and *Tcf21* (19) (Figure 1D). We noted that clusters 1 and 2 are differentiated from cluster 0 by expression of AP-1 transcription factor genes (*Fos*, *Jun*, *Fosb*, and *Junb*), regulation of proliferation (*Btg2*, *Cyr61*, and *Id3*), and stress response genes (*Atf3*, *Dusp1*, *Gadd45b*, and *Ppp1r15a*) (Supplemental Figure 5). Cluster 2 was enriched for genes associated with cellular stress response (*Maff*, *Nr4a2*, and *Klf4*), and *Tgfb* response (*Bambi*), among others. Bleomycin-derived LipoFB cluster 6 was similar to cluster 0 for LipoFB marker expression (Supplemental Figure 5). Ultimately, based on the similarities between clusters 0 and 6, we view these as the canonical LipoFB clusters for naive and fibrotic lung, respectively.

**Figure 2:**
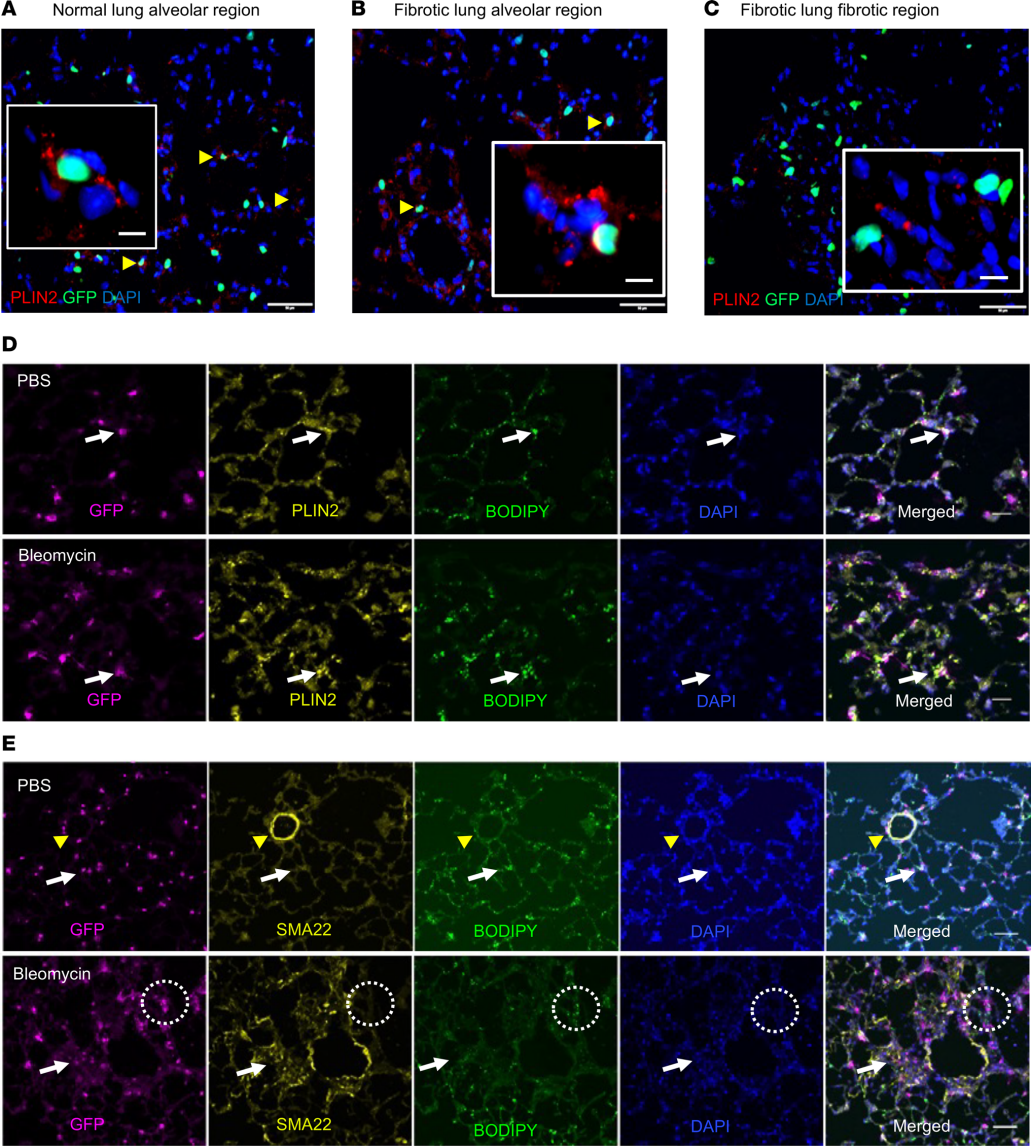
Characterization of gene markers in normal and fibrotic *Pdgfra^GFP^* mouse lung. (**A**–**C**) Localization of PLIN2 expression by immunofluorescent staining in normal (**A**) and fibrotic (**B** and **C**) lung. Yellow arrowheads show GFP*^+^* cells with adjacent PLIN2 expression. (**D**) Localization of GFP, PLIN2, BODIPY^+^ lipid droplets, and DAPI in normal (PBS) and fibrotic (bleomycin) lung. Arrows indicate area of colocalization of all markers. (**E**) Localization of GFP, SMA22, BODIPY^+^ lipid droplets, and DAPI in normal (PBS) and fibrotic (bleomycin) lung. Arrows indicate a region of high SMA22 expression and low lipid droplet accumulation; the dotted circle highlights an area of low SMA22 expression and high lipid droplet accumulation. Yellow arrowheads show SMA22^+^ smooth muscle cells. Scale bars: 50 μm (**A**–**C**), 10 μm (**A**–**C**, insets), and 20 μm (**D** and **E**).

Distribution of the canonical LipoFB gene *Plin2* across clusters showed highest expression in normal cluster 0 and fibrotic cluster 6. The LipoFB phenotype in naive and fibrotic lungs was validated by immunofluorescent staining for PLIN2 (also known as ADRP) (Figure 2, A–C). PLIN2 expression was localized in alveolar regions with GFP^+^ cells in normal (Figure 2A) and fibrotic (Figure 2B) lungs (arrowheads and inserts). Little PLIN2 expression was found in fibrotic regions of the lung (Figure 2C, inset), in agreement with the low level of gene expression in cluster 9. We also found that lipid droplets colocalized with PLIN2 in GFP^+^ FBs in PBS-exposed lungs and in alveolar regions of bleomycin-treated lungs (Figure 2D; PBS top panel, bleomycin lower panel, arrows). We evaluated expression of SMA22 (transgelin, TAGLN; Figure 2E), a canonical marker of MyoFB identified in our gene expression analysis (Figure 1D). As expected, SMA22 was expressed predominantly in smooth muscle cells in normal lung (Figure 2E; PBS, arrowheads), with little expression in alveolar regions (arrows), while there was increased expression in fibrotic lung (Figure 2E; bleomycin, arrows). In addition, while lipid droplets are strongly evident in SMA22-negative regions of normal and injured lung, there was a significant decrease in lipid droplet staining in SMA22-positive regions in fibrotic lung (Figure 2E; arrows, dotted circles). Taken together, these data support our gene clustering identification of LipoFB and MyoFB cell populations and suggest that clusters 0 and 6 reliably represent gene profiles of LipoFBs in normal and injured lungs.

### Identification of a common LipoFB gene signature between naive and fibrotic LipoFBs.

**Figure 3:**
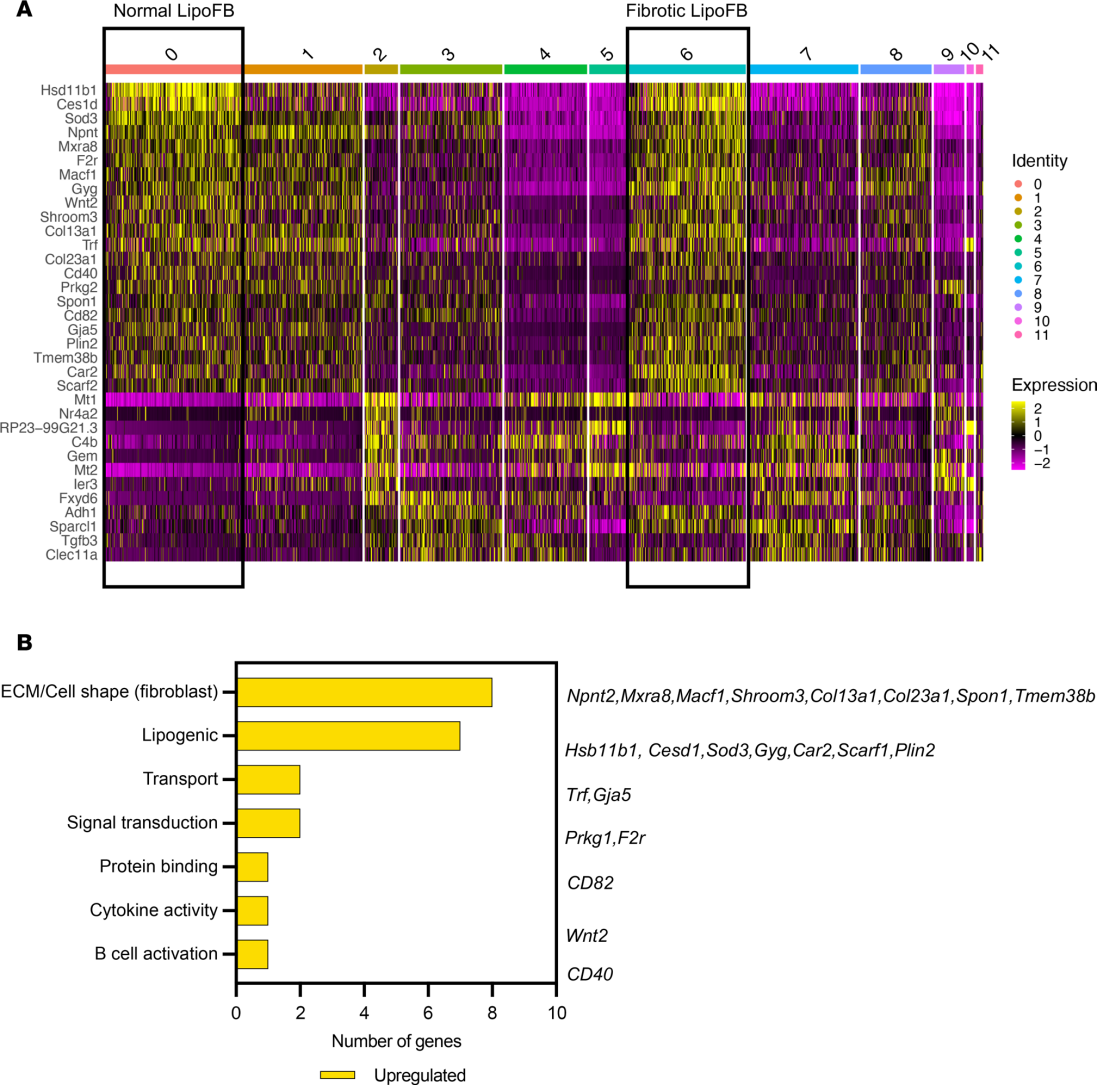
A unique shared transcriptomic signature supports LipoFB identity in normal and fibrotic lung. (**A**) Heatmap representation of up- and downregulated genes across all clusters showing conserved expression between normal (cluster 0, boxed) and fibrotic (cluster 6, boxed) *Pdgfra*^+^ LipoFBs. (**B**) Bar chart of 22 upregulated shared genes between clusters 0 and 6 indicating functional categories and signature genes in each category.

Next, we specifically asked the question whether there are genes that remain relatively unchanged between clusters 0 (naive canonical LipoFBs) and 6 (injured canonical LipoFBs) to identify a possible shared signature that can serve to maintain the LipoFB phenotype between normal and injured states. Using differential gene expression analysis on cluster data (Supplemental Table 2), we identified a unique transcriptomic signature of 22 upregulated genes shared between normal and injured LipoFBs (Figure 3, A and B). These included canonical LipoFB genes, e.g., *Plin2*, *Wnt2a*, and *Col13a1* (16, 17), 7 genes annotated to lipogenic functions, and 8 genes associated with FB signatures (Figure 3B). These genes were strongly downregulated in MyoFB-specific cluster 9 (Figure 3A). As LipoFB and MyoFB signatures are mutually exclusive (16), these genes can therefore be defined as core genes of LipoFBs.

### Transitional gain of fibrogenic and loss of lipogenic function in injured lung FBs.

**Figure 4:**
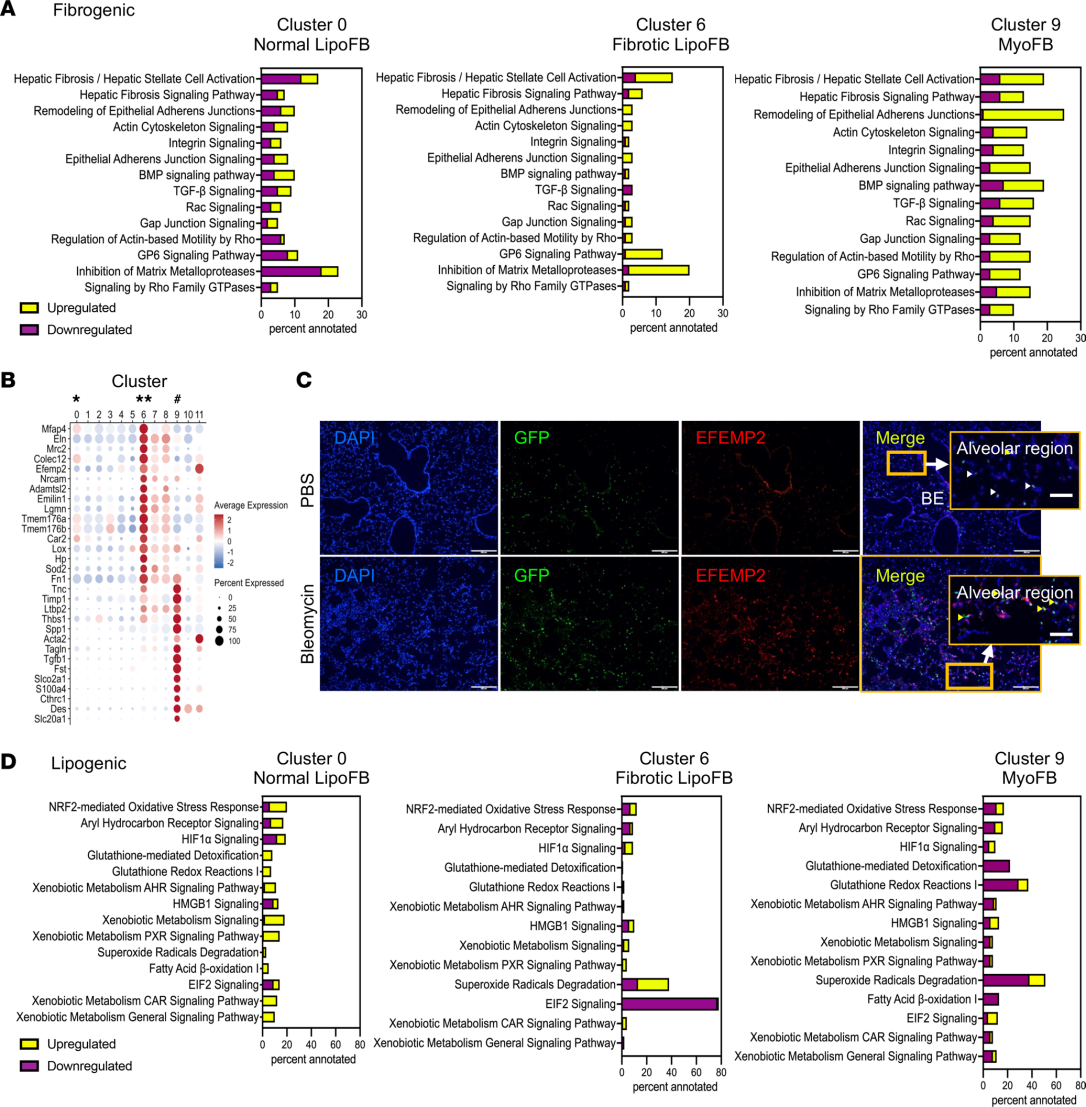
*Pdgfra^+^* LipoFBs acquire a fibrotic phenotype following injury, with attenuation of lipogenic pathways. (**A**) Canonical pathway profiling of fibrogenic pathways between normal LipoFBs (cluster 0), fibrotic LipoFBs (cluster 6), and activated MyoFBs (cluster 9). Yellow indicates upregulated, purple downregulated. (**B**) Dot plot of stress fiber– and ECM-related genes. * = cluster 0, normal LipoFB; ** = cluster 6, injured LipoFB; # = cluster 9, MyoFB. (**C**) Localization of EFEMP2 and GFP in normal and injured lung; insets show alveolar regions. Yellow arrowheads point to regions of colocalization and white arrowheads show GFP with no EFEMP2 coexpression. Scale bars: 200 μm and 50 μm (insets). (**D**) Canonical pathway profiling of lipogenic pathways between normal LipoFBs (cluster 0), fibrotic LipoFBs (cluster 6), and activated MyoFBs (cluster 9).

To understand how injury affects the response of *Pdgfra^+^* FBs to injury, we compared normal and injured LipoFBs and MyoFBs utilizing Ingenuity Pathway Analysis. Activation and transdifferentiation of *Pdgfra^+^* LipoFBs to MyoFBs after bleomycin-induced injury has been previously described (13). In our studies, cluster 9 was identified as MyoFBs, which represent the most activated FBs. We therefore used marker genes in cluster 9 to develop a “fibrogenic profile,” which included pathways such as hepatic fibrosis signaling, actin cytoskeleton signaling, *Tgfb* signaling, and inhibition of matrix metalloproteinases (Figure 4A). As expected, these pathways were downregulated in normal LipoFBs (cluster 0, Figure 4A). However, in injured LipoFBs (cluster 6, Figure 4A), there was upregulation of many of these pathways, including inhibition of matrix metalloproteases and G6 signaling pathways. These data suggest that bleomycin injury results in a spectrum of MyoFB-like activation in LipoFBs.

We identified a series of genes enriched in injured LipoFBs (cluster 6) that annotated to stress fiber formation, actin cytoskeleton, and ECM, including *Mfap4*, *Eln*, *Efemp2*, and *Adamtsl2* (Figure 4B). EFEMP2 (EGF-containing fibulin ECM protein 2), an ECM protein important for the formation of elastic fibers (20), is largely absent in normal alveolar regions (Figure 4C; PBS, upper panel, inset and arrowheads). However, immunofluorescence for EFEMP2 shows high expression in alveolar regions containing GFP^+^ FBs in fibrotic lung (Figure 4C; lower panel, bleomycin, inset and yellow arrowheads. White arrowheads point to areas of GFP expression in the absence of associated EFEMP2). *Lox* (lysyl oxidase), which was also enriched in this group of genes (Figure 4B), has been shown to facilitate stress fiber formation in conjunction with *Efemp2* (21–23). Other enzymes in this group include *Car2*, *Hp*, and *Sod2,* the latter 2 of which have roles in regulating the effects of oxidative stress (24, 25).

Examining the lipogenic profile shows that in normal lung LipoFBs, the strongest annotation was to oxidative stress response, xenobiotic metabolism, and detoxification (Figure 4D). These pathways were downregulated in MyoFBs and were downregulated or largely not present in injured LipoFBs. These data suggest that LipoFBs in injured lung experience attenuation of the lipogenic phenotype while concomitantly acquiring characteristics of MyoFBs in fibrotic lung.

### Normal LipoFBs can attain regenerative or pathologic lineage trajectories after injury.

**Figure 5:**
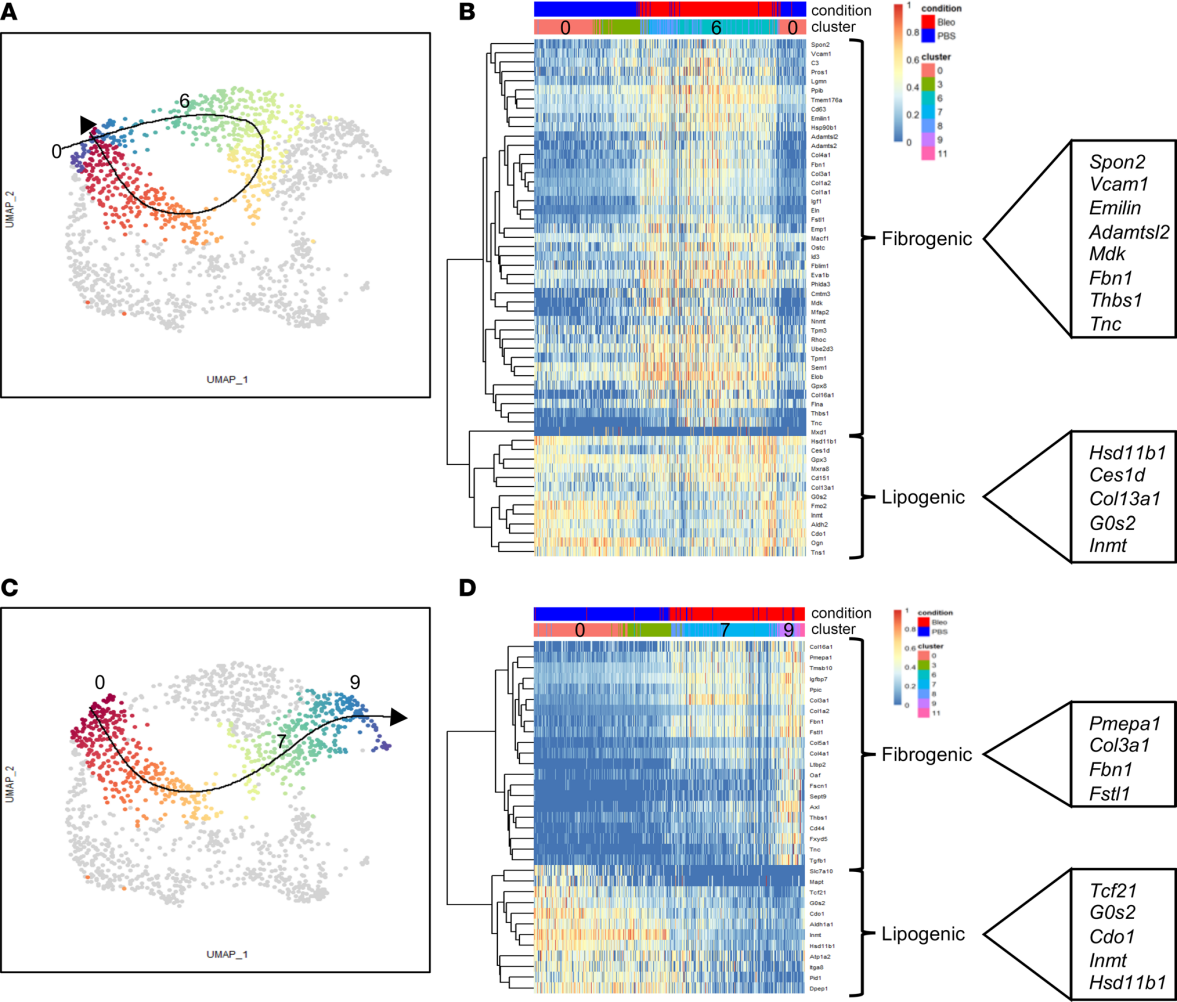
LipoFBs undergo regenerative or pathologic trajectories following bleomycin-induced fibrosis. (**A**) Pseudotime analysis using normal LipoFBs (cluster 0) as the origin, with a loop trajectory through fibrotic LipoFBs (cluster 6) back to normal LipoFBs. (**B**) Heatmap of fibrogenic and lipogenic gene transition in the loop regenerative trajectory. (**C**) Pseudotime analysis using normal LipoFBs (cluster 0) as the origin, with a linear trajectory progression through cluster 7 and terminating at MyoFBs (cluster 9). (**D**) Heatmap of fibrogenic and lipogenic gene transition in the linear pathologic trajectory.

Pseudotime analysis with normal LipoFBs (cluster 0) as the point of origin, identified trajectories to both fibrotic LipoFBs (cluster 6) and MyoFBs (cluster 9) (Figure 5, A and C). Normal LipoFBs (cluster 0) can transition to LipoFBs from fibrotic lung (cluster 6) and loop back to normal (Figure 5A). Gene expression changes in this loop trajectory were associated with upregulation of wound response genes in the injured fibrotic LipoFBs (cluster 6), while the lipogenic signature remained largely unchanged (Figure 5B). These data suggest an injury-induced activation status with the potential to revert to normal as long as the lipogenic function is remained. Genes specifically activated in this wound-responsive LipoFB cluster are associated with ECM organization and wound healing (e.g., *Spon2*, *Vcam1*, *Emilin1*, *Adamtsl2*, *Mdk*, *Fbn1*, *Thbs1*, and *Tnc*) (Figure 5B). Upregulation of these genes suggest activation of a regenerative MatrixFB function.

The second trajectory originated in normal LipoFBs (cluster 0) and progressed through injury-activated LipoFBs (cluster 7), to end in MyoFBs (cluster 9) (Figure 4C). Gene expression changes in this directional trajectory show acquisition of fibrogenic genes and concomitant loss of lipogenic genes as the trajectory progresses from normal LipoFBs via transitional LipoFBs (cluster 7) to MyoFBs (cluster 9) (Figure 5D). Genes especially upregulated in the transitional LipoFBs are associated with integrin binding, ECM tensile strength, and TGF-β signaling (*Fbn1*, *Fstl1*, *Pmepa1*, and *Col3a1*), suggesting a response to increased TGF-β signaling. Thus, our analysis suggests a plasticity in the injury response of LipoFBs, which can assume genetic profiles supporting either homeostasis or fibrosis development.

### Repetitive bleomycin exposure induces a transcriptomic profile similar to that of a single dose on day 14.

To confirm that the transcriptomic profile described above is a generalized phenomenon in the fibrotic response, we subjected *Pdgfra^GFP^* mice to repetitive bleomycin exposure and conducted scRNA-Seq on GFP^+^ FBs 14 days after the last of 3 once-weekly doses of bleomycin or PBS (Supplemental Figure 6A). From this analysis, 25 clusters were found (Supplemental Figure 6B and Supplemental Table 3; quality control data available in Supplemental Table 1) and, as shown in the single-dose model (Figure 1C), the clusters exhibited a spatial distribution across PBS or bleomycin (Supplemental Figure 6, C and D). To identify known populations, we used the same signature genes as shown in Figure 1D to generate a dot plot to highlight normal (clusters 0–3 and 12) and injured LipoFBs (cluster 6), MyoFBs (cluster 18), and MatrixFBs (clusters 7, 8, and 15) (Supplemental Figure 7, A and B). In addition, PMP and MEFB populations were identified in clusters 16 and 25, respectively. The LipoFB signature gene set was retained in injured and normal LipoFBs, as shown in Figure 3, A and B, and Supplemental Figure 7C. These data demonstrate that bleomycin induces a core injury profile, generating a population of injured LipoFBs that contribute to the pathogenesis of fibrosis.

### Data mining mouse and human scRNA-Seq data sets reveals LipoFB populations associated with normal and fibrotic lung.

**Figure 6:**
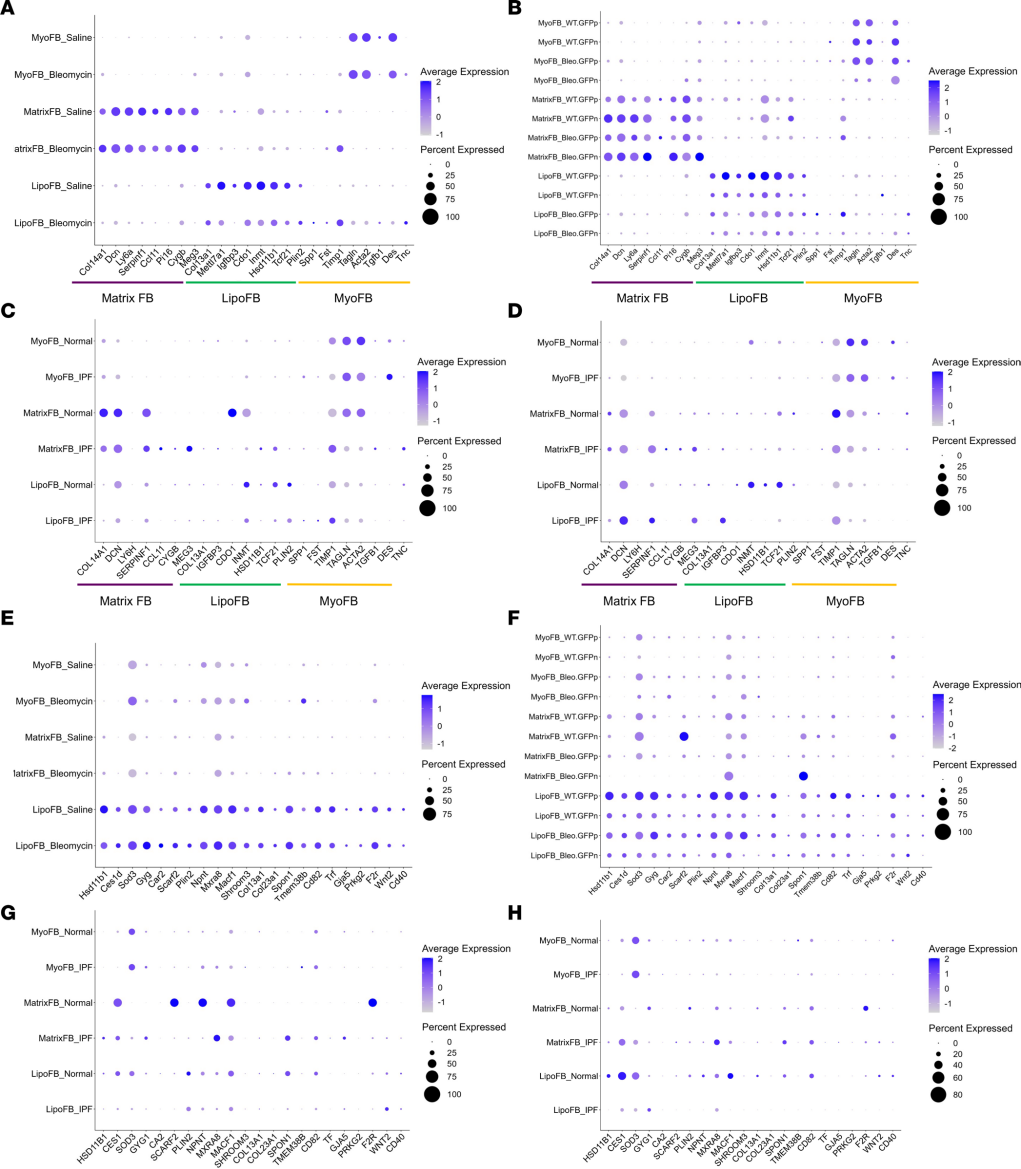
Fibroblast subpopulations in normal and fibrotic lungs of humans and mice revealed by scRNA-Seq analyses. (**A**) Fibroblast populations in 4 bleomycin-induced and 4 saline control lung tissues on day 11 from GEO GSE129605. (**B**) Fibroblast populations on day 14: scRNA-Seq fibrosis and wild-type control data from 2 *Col1a1^GFP^* reporter mice treated with bleomycin (GEO GSE132771). (**C**) Fibroblast populations in human lung tissue: scRNA-Seq data from 8 normal lung transplant donors and 4 IPF patients (GEO GSE122960). (**D**) Fibroblast populations in human scRNA-Seq lung data from 3 normal individuals and 3 IPF patients (GEO GSE132771). For panels **A**–**D**, the dot plots display the expression patterns of fibroblast subtypes using top markers from Figure 1D. (**E**–**H**) The same data sets (**A**–**D**, respectively) queried with the shared LipoFB gene from Figure 3. IPF, idiopathic pulmonary fibrosis; MatrixFB, matrix fibroblast; LipoFB, lipofibroblast; MyoFB, myofibroblast; PMP, proliferating mesenchymal progenitors; ME, mesothelial cell.

In addition to the repetitive bleomycin exposure analysis described above, we utilized published scRNA-Seq databases for both mouse (GSE129605, ref. 26; GSE132771, ref. 27) and human (GSE122960, ref. 28; GSE132771, ref. 27) to ask the question whether our data could be used to identify LipoFB populations in normal and fibrotic mouse and human lungs (Figure 6, A–D, and Supplemental Table 4). We utilized the top gene markers for MatrixFBs, LipoFBs, and MyoFBs (Figure 1D) to annotate the FB subset data, as described in Methods. Using this approach, we identified distinct MatrixFB, LipoFB, and MyoFB populations in both mouse data sets, with LipoFBs segregating between bleomycin and PBS treatment groups, in agreement with our findings (Figure 6, A and B). In the 2 human data sets (Figure 6, C and D), all 3 subpopulations were identified, but LipoFB populations were only found in normal human lung FBs, and not in FBs from end-stage IPF lungs. We also queried the same data sets shown in Figure 6, A–D with the shared LipoFB signature described above in Figure 3. As can be seen in Figure 6, E and F, the shared gene signature was similar between both control and bleomycin-treated mouse lung FBs, consistent with our findings in the *Pdgfra^GFP^* reporter mouse. The shared signature was also evident in normal human lung FBs (Figure 6, G–H), while expression was attenuated in IPF lung FBs, highlighting the overall loss of the LipoFB population in human IPF. This indicates that in the case of advanced fibrotic disease in humans, the LipoFB phenotype is lost or severely diminished below detection capability, most likely due to transdifferentiation into activated MyoFBs. This data mining strategy demonstrates that the transcriptomic signatures found in controlled and well-studied animal models can be used to identify similar populations in progressive and end-stage human disease data sets.

### Multiomics profiling demonstrates shared proteomic and transcriptomic patterns in normal and fibrotic Pdgfra^+^ FBs.

We next undertook experiments to integrate our transcriptomic data with proteomic data to determine whether the unique gene expression patterns in normal and injured FBs correlated with functional protein expression. To do this, we isolated protein from *GFP^+^* FBs obtained from PBS- or bleomycin-dosed mice, following the model shown in Figure 1A. A total of 4 biological replicates were conducted, and protein was isolated and assessed by liquid chromatography–tandem mass spectrometry (LC-MS/MS). Principal component analysis (PCA) showed good separation of bleomycin and PBS samples (Supplemental Figure 8A). A total of 2,898 proteins were identified with high or medium confidence using Proteome Discover (Thermo Fisher Scientific, OPTON-31014) and Spectrum Mill (https://proteomics.broadinstitute.org/millhome.html). Of these, 394 had at least a 2-fold difference in abundance, with *P* values less than 0.05 between conditions (Supplemental Figure 8B). Robust reproducibility between biological replicates for specific proteins is shown in Supplemental Figure 8, C and D (upregulated in bleomycin and PBS, respectively). A heatmap of proteins in normal and fibrogenic *Pdgfra^+^* FBs demonstrates the inverse relationship between normal and fibrogenic expression patterns (Supplemental Figure 9A).

Protein expression data were analyzed using normalized abundance values from Proteome Discoverer and Spectrum Mill, transformed with log_2_(*x* + 1), and then median normalized. Keratin proteins were removed and detected proteins (2,777 total) were defined by having values in at least 2 replicates of at least one sample group. This generated a list of up- and downregulated proteins in bleomycin versus PBS and determines the relative protein expression changes in fibrogenic *Pdgfra*^+^ FBs (Supplemental Table 5). The MSigDB Canonical Pathway gene set (https://www.gsea-msigdb.org/gsea/msigdb/collections.jsp) was used to evaluate statistically significant (*P* < 0.05) upregulated and downregulated proteins with greater than or equal to 2-fold change. Proteins upregulated in fibrogenic *Pdgfra*^+^ FBs were all related to structural proteins associated with fibrosis-mediated remodeling, e.g., biological processes associated with collagen biosynthesis, collagen formation, and ECM organization (Supplemental Figure 9B and Supplemental Table 6, enriched), all related to structural functions associated with fibrosis-mediated remodeling. Proteins exhibiting downregulation in fibrogenic FBs largely annotated to metabolic processes, including oxidation, *Nrf2* pathway, and cytochrome p450 metabolism (Supplemental Figure 9C and Supplemental Table 6, depleted). The biological processes in the protein data broadly reflect fibrogenesis concomitant with decreased metabolic stress–response pathways.

**Figure 7:**
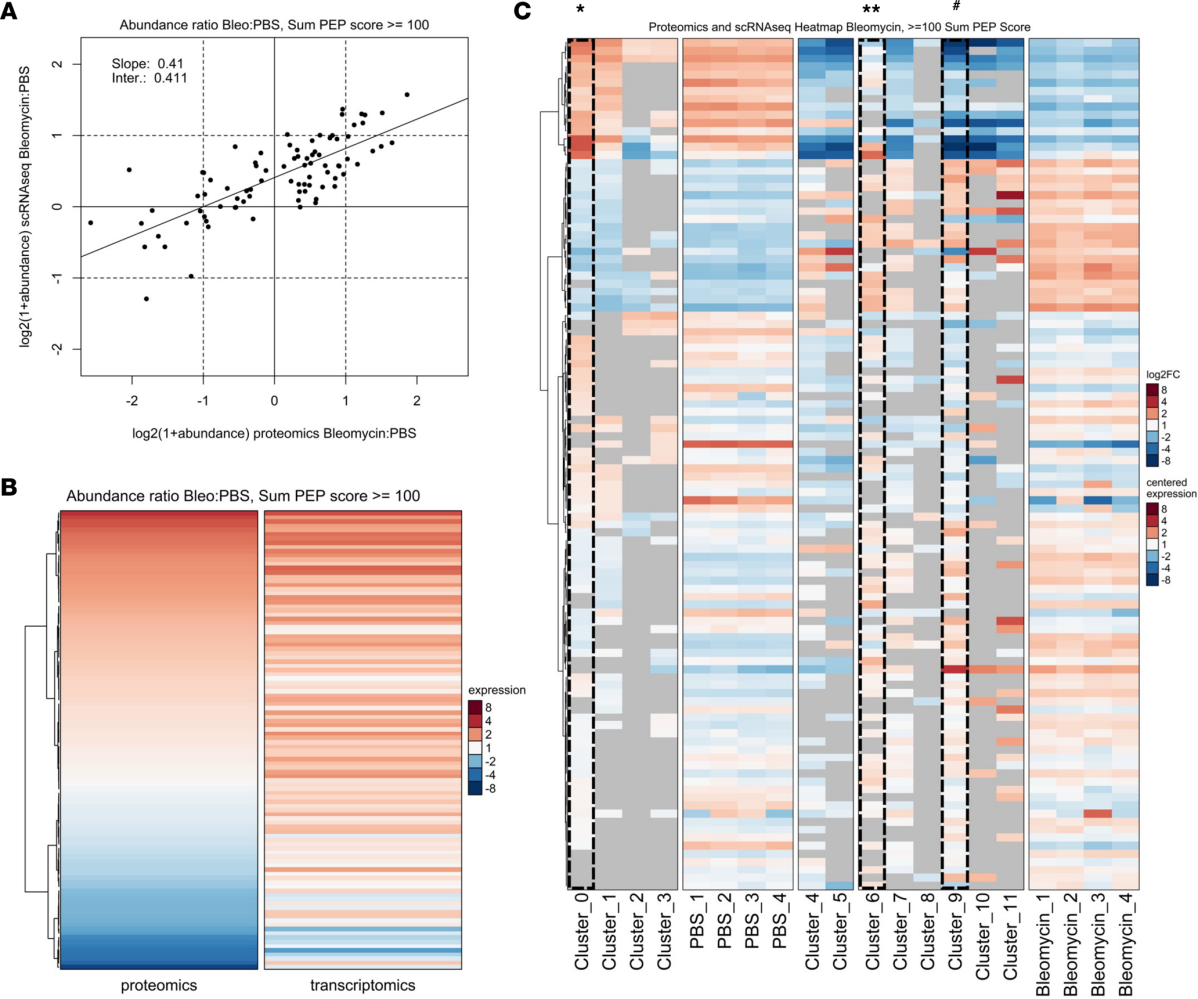
Proteomic and transcriptomic multiomics analyses reveal directional lipogenic and fibrogenic expression patterns in normal and injured *Pdgfra^+^* fibroblasts. High confidence identifications (≥100 sum posterior error probability [PEP] score). (**A**) Scatterplot of log(bleomycin/PBS ratio) of protein vs. RNA data. (**B**) Heatmap of log(bleomycin/PBS ratio) of protein vs. RNA data. (**C**) Heatmap of protein samples (PBS 1–4 and bleomycin 1–4) and RNA clusters (clusters 0–11) from normal and fibrogenic *Pdgfra^+^* fibroblasts. Protein columns are centered log_2_-transformed data. RNA columns are log_2_(fold change) [log2(FC)] data. All columns are labeled with normal fold change values. Columns have been ordered to emphasize correlations. * = cluster 0, normal LipoFB; ** = cluster 6, injured LipoFB; # = cluster 9, MyoFB.

A comparison analysis between the scRNA-Seq and proteomic data sets was conducted to determine whether the integration of the 2 data sets would yield additional insights into the biology of fibrogenic lung FBs, similar to the approach taken by Du et al. (29). Directional and abundance relationships of the transcriptomic and proteomic data show a positive relationship between protein and RNA expression, with 76% of protein and RNA directionally related (Figure 7, A and B). The heatmap reflects that most genes/proteins had a similar response or at least a similar directionality in the bleomycin/PBS ratio (Figure 7B).

We then correlated the proteomic data with the transcriptomic data at the level of the individual clusters from the transcriptomic profiling of normal and fibrogenic *Pdgfra^+^* FBs. The left panels of Figure 7C show how the gene expression in the PBS-derived transcriptomic clusters (clusters 0, 1, 2, and 3) compared to protein expression in the 4 PBS biological replicates; 75% of the genes in these 2 groups share regulation directionality. Upregulated genes that are part of the LipoFB signature, such as *Hsd11b1*, *Ces1d*, *Selenbp1*, and *Gstm2* were also upregulated at the protein expression level, and the pattern of gene expression correlated positively with that of protein expression in normal *Pdgfra^+^* FBs (Figure 7C). Similarly, clusters derived from bleomycin (6–11) show matching patterns of up- and downregulated expression between the RNA and protein data (also 75% matching directionality). Because cluster 6, which is the injured LipoFB population, was intermediate between PBS and bleomycin, we were interested in further defining the protein/gene expression differences between normal and injured LipoFBs. To do this, we analyzed the different expression relationships between clusters 0 and 6 using Gene Ontology (GO) (http://geneontology.org/). We found that biological processes associated with uniquely upregulated expression in injured LipoFBs included responses to endoplasmic reticulum stress, protein folding, and cytoskeleton-related processes (Supplemental Table 8), consistent with fibrogenic changes. In addition, biological processes annotated to downregulated expression in injured LipoFBs annotated to metabolic processes, consistent with attenuation of the lipogenic phenotype. Finally, we prepared dot plots of genes/proteins identified as uniquely upregulated in injured LipoFBs or downregulated in injured LipoFBs relative to normal (Supplemental Figure 10, A and B; upregulated in 6 and downregulated in 6, respectively). These plots again show the distinct pattens between PBS- versus bleomycin-derived cells, but also demonstrate that injured LipoFBs match directionality with MyoFBs (cluster 9), underlining the fibrogenic changes these FBs undergo with injury and supporting the pathway analysis that showed that injured LipoFBs acquire a fibrogenic phenotype at the expense of lipogenic pathways (Figure 4) by demonstrating that this occurs at the protein level as well. Taken together, the multiomic analyses demonstrate a profound *Pdgfra^+^* FB response to injury and fibrogenesis.

### Fibrogenesis peaks on day 14 after bleomycin and correlates with human IPF bulk transcriptomics signature.

**Figure 8:**
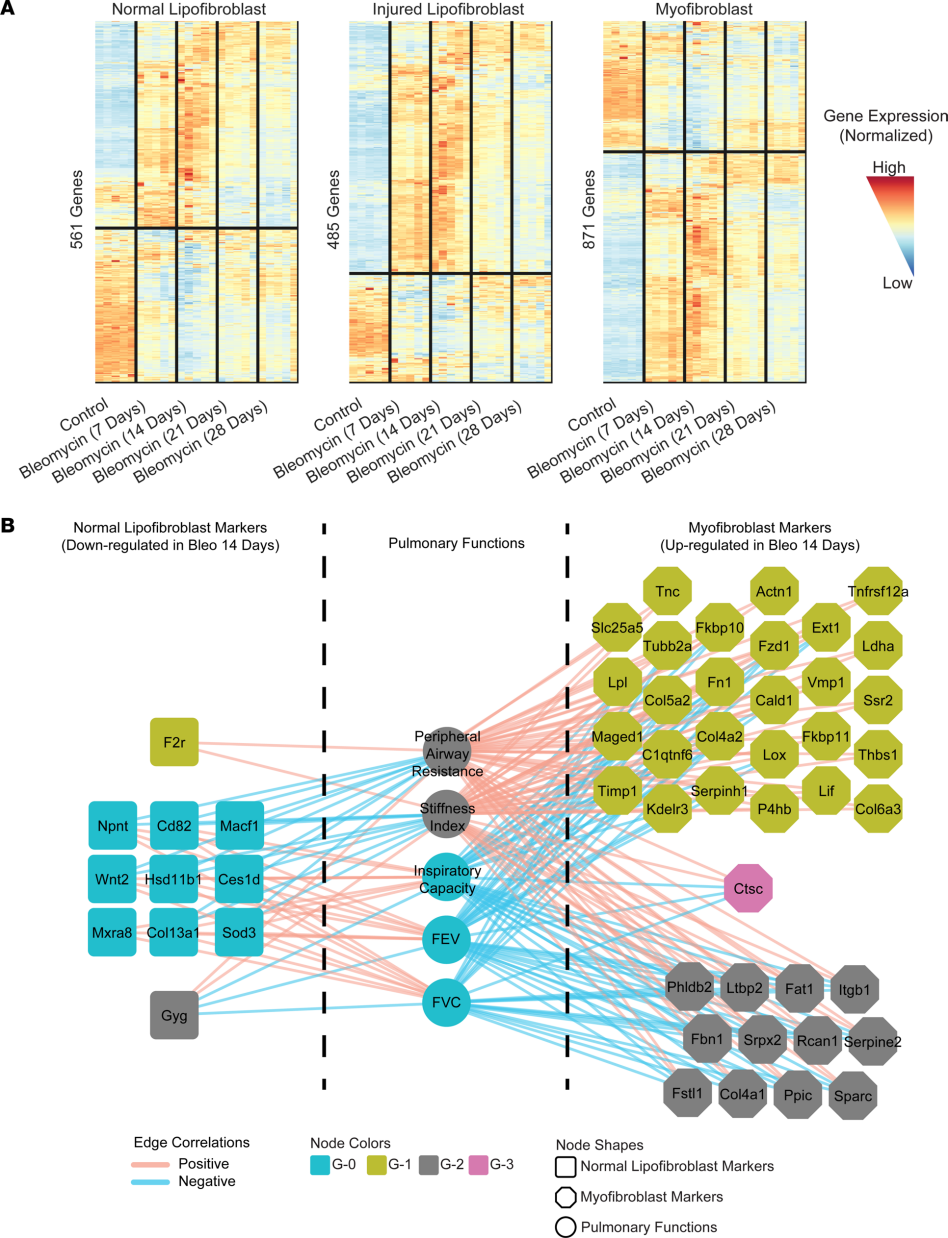
Correlation of normal and fibrogenic fibroblast marker genes with pulmonary fibrosis and disease progression. (**A**) Normalized TPM expression of normal lipofibroblast (cluster 0), injured lipofibroblast (cluster 6), and myofibroblast (cluster 9) markers in bleomycin-induced pulmonary fibrosis bulk lung transcriptomic data from the Mouse Lung Fibrosis Atlas. (**B**) Normal lipofibroblast and myofibroblast marker genes in the gene coexpression network from the Mouse Lung Fibrosis Atlas.

It has been established that fibrogenesis in the lung is high 2 weeks after bleomycin-induced injury (30–32). To confirm the representative value of our chosen time point, as well as the relevance of our discovered gene signatures to human disease, we reanalyzed our scRNA-Seq data using the Mouse Lung Fibrosis Atlas (https://niaaa.nih.gov/mouselungfibrosisatlas) (33) to correlate our data with mouse pulmonary fibrosis (PF) transcriptomics and pulmonary function. In the Mouse Lung Fibrosis Atlas, the authors identified PF progressive genes and assessed their translatability in human IPF patients in which the transcriptomic signature from fibrotic lungs on day 14 after bleomycin in mice resembles IPF patients’ lungs (33). Reanalysis of our data set clearly demonstrated that the up- and downregulation of LipoFB and MyoFB marker genes peaked 14 days after bleomycin compared with controls in the bulk lung transcriptome (Figure 8A), thus supporting our time point selection for the scRNA-Seq analysis. To further understand the role of the FB marker genes in the progression of IPF and their association with pulmonary function, we retrieved the gene-coexpression network from the Mouse Lung Fibrosis Atlas (33). Using this network, we found that the normal LipoFB markers were mainly located in the G-0 subnetwork (Figure 8B). These normal LipoFB markers were positively correlated with forced expiratory volume, forced vital capacity, and inspiratory capacity, and negatively correlated with peripheral airway resistance and stiffness index. The normal LipoFB markers showed significant downregulation 14 days after bleomycin, while MyoFB markers were in subnetworks G-1 and G-2 and showed the opposite expression and correlation trends (Figure 8B). Since G-1 and G-2 were previously identified as the driver subnetworks of PF progression in mice and IPF patients (33), these analyses confirm the relevance of our fibrogenic gene sets as potential targets in human IPF.

### Fibrogenic FBs support alveolar epithelial cell differentiation but induce changes in organoid morphology.

**Figure 9:**
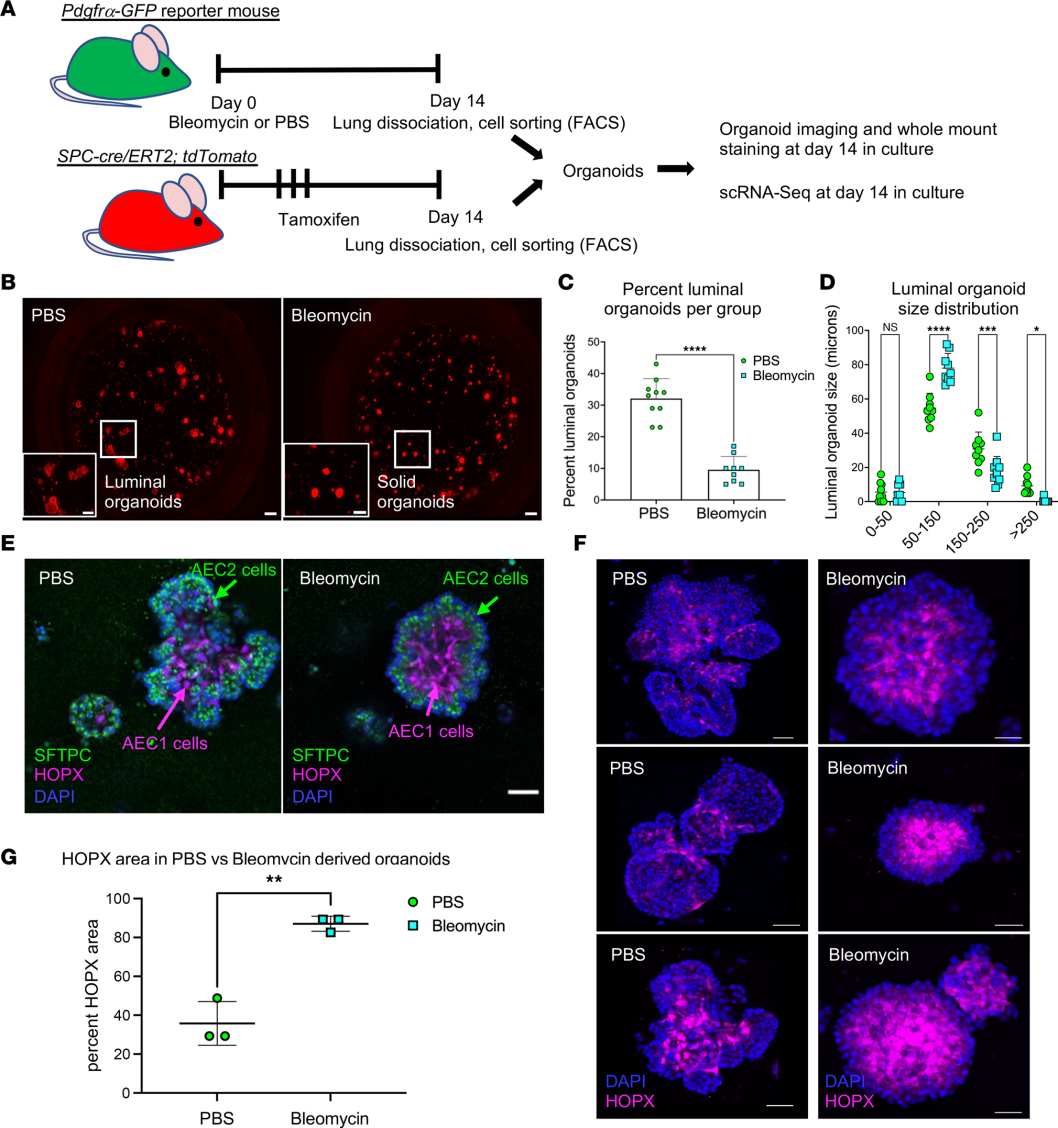
Alveolosphere culture reveals morphological differences in organoids derived from fibrogenic *Pdgfra*^+^ fibroblasts versus normal fibroblasts. (**A**) Diagram of experimental workflow. (**B**) Representative epifluorescence images of alveolospheres on day 14 of culture (PBS on the left and bleomycin on the right). Scale bars: 500 μm. Insets show luminal organoid (PBS) and solid organoid (bleomycin) morphology. Scale bars: 250 μm. (**C**) Measurement of percentage luminal organoids per treatment group. (**D**) Luminal organoid size distribution per treatment group. Organoid morphology and size distribution were measured from a total of 9 individual cultures per treatment. Data shown here are representative of 2 independent experiments. (**E**) Whole-mount staining and confocal imaging showing localization of SFTPC (marks AEC2s) and HOPX (marks AEC1s) in alveolospheres derived from PBS-derived (left panel) and bleomycin-derived (right panel) *Pdgfra*^+^ fibroblasts. Scale bar: 50 μm. (**F**) Representative luminal (PBS) and condensed (bleomycin) organoids stained for HOPX and with DAPI used for determination of percentage HOPX area between cluster morphologies (total of 3 organoids measured per group). Scale bars: 50 μm. (**G**) Percentage HOPX^+^ area between PBS-treated (normal) and bleomycin-treated (fibrogenic) fibroblast–supported alveolospheres. *P < 0.05; **P < 0.001; ****P < 0.0001 by 2-tailed Student’s *t* test (**C** and **G**) or 2-way ANOVA with Šidák’s correction for multiple comparisons (**D**).

In vitro organoid cultures are a well-established tool to interrogate the role of *Pdgfra^+^* FBs in organoid formation and type 2 alveolar epithelial cell (AEC2) into type 2 alveolar epithelial cell (AEC1) differentiation (9, 34, 35). GFP^+^ cells from bleomycin- or PBS-treated adult mice were harvested 2 weeks after treatment and cultured with normal SFTPC-tomato–positive AEC2s isolated from adult mouse lungs (Figure 9A). There was no significant difference in aggregate colony size or number (data not shown). However, there was a significantly reduced number of colonies with luminal morphology in organoids that developed in coculture with bleomycin-injured fibrogenic *Pdgfra^+^* FBs (Figure 9B, insets). The few luminal organoids found in the bleomycin-induced organoids were both significantly fewer in number (Figure 9C) and significantly smaller (50–150 μm) as compared with PBS (150–250 and >250 μm; Figure 9D), indicating a restricted growth of this organoid type. Fluorescence images of each well are in Supplemental Figure 11, A and B (PBS and bleomycin, respectively).

After 2 weeks in organoid cultures, expression of SFTPC (AEC2) and HOPX (AEC1) was assessed by immunofluorescence. No difference in AEC2 self-renewal and differentiation into AEC1s was observed (Figure 9E). Arrangement of these 2 populations with SFTPC^+^ cells on the outer surface of the organoid and HOPX^+^ cells on the interior was comparable (9) (Figure 9E). To better examine the morphological differences between the 2 groups, a montage of 6 sequential *Z*-slices were prepared of a PBS-derived and a bleomycin-derived colony (Supplemental Figure 12; PBS upper panels, bleomycin lower panels). These images show that organoids grown with PBS-control GFP*^+^* FBs tend to have a lobed or irregular formation that exists throughout the organoid, while those from bleomycin-derived FBs have a regular spherical shape with densely compacted AEC1s filling the interior of the sphere. The stratification of AEC2s and AEC1s was not different between the 2 culture conditions. However, we found that the percentage area of HOPX^+^ cells was significantly higher in the densely compacted organoids in cultures from bleomycin-derived GFP*^+^* FBs compared with the luminal organoids typical of the PBS-derived FB cultures (Figure 9, F and G). Ultrastructural imaging revealed that organoids from normal FBs had numerous intracytoplasmic vesicles containing amorphous material and coalescing fat droplets, which occupied the bulk of the cytoplasm. In contrast, few vesicles were present in the organoids from fibrogenic FBs, with only rare fat droplets and an absence of dilated rough endoplasmic reticulum (Supplemental Figure 13). Taken together, these data suggest that fibrogenic *Pdgfra^+^* FBs can induce ultrastructural changes within AEC2s in organoid culture. In summary, LipoFBs from fibrotic lung can promote basic AEC2 self-renewal and differentiation, but do not support growth of organoids that form a mature alveolar lumen, suggesting a lack of supportive matrix derived from fibrogenic FBs.

### Transitional AEC2 populations form in normal and fibrogenic organoids.

**Figure 10:**
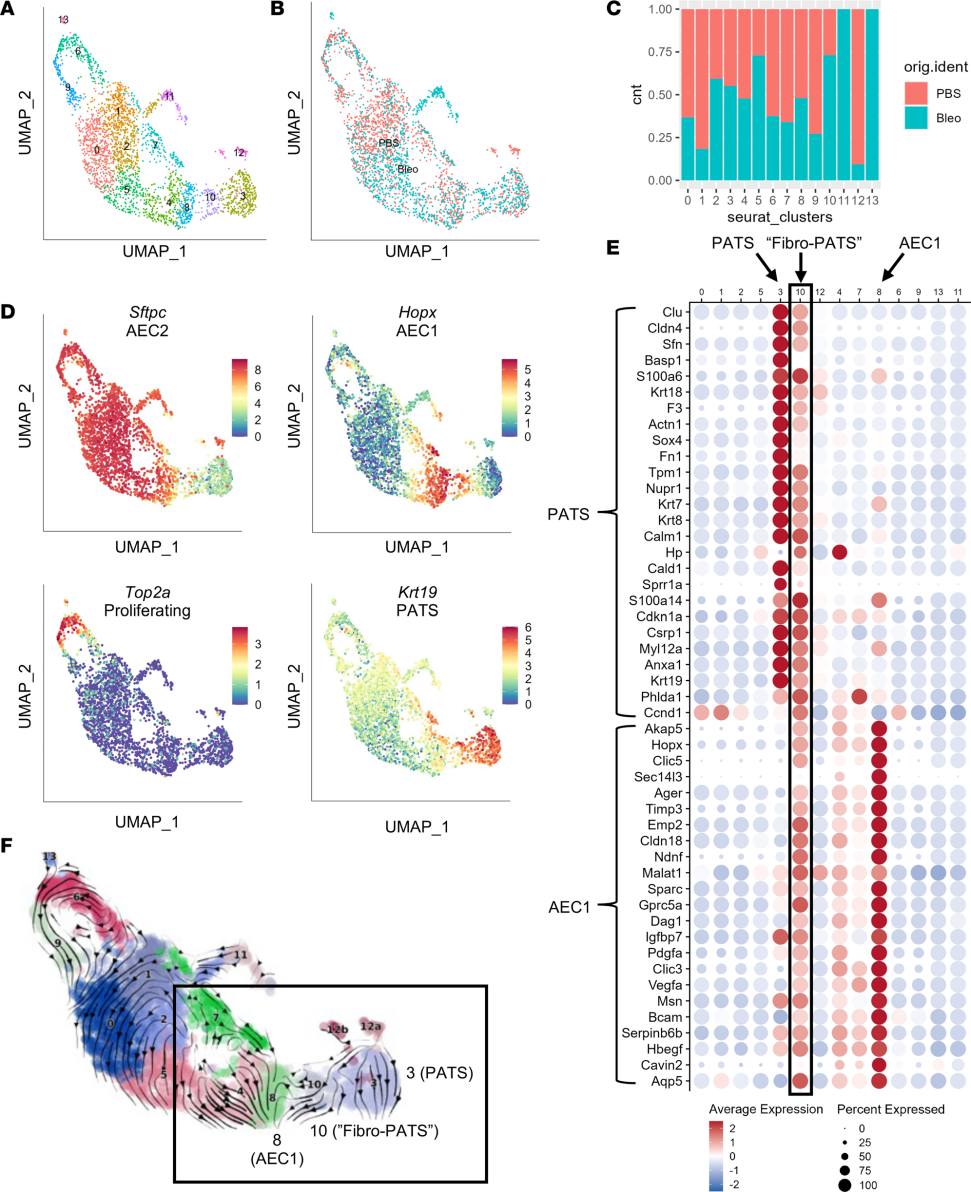
Transcriptomic analysis of epithelial cells in alveolosphere cultures grown from normal and fibrogenic *Pdgfra*^+^ fibroblasts revealed a fibrogenesis-specific AEC2 transitional population. (**A**) UMAP of 12 individual clusters of organoid-derived epithelial cells. (**B**) UMAP showing split distribution between PBS (normal) and bleomycin (fibrogenic) cultures. (**C**) Cell proportions between treatment groups. (**D**) Scatter plots showing cluster localization of AEC2 (*Sftpc*), AEC1 (*Hop*x), proliferating alveolar epithelial cells (*Top2a*), and PATS (pre-alveolar type 1 transitional state; *Krt19*). (**E**) Dot plot of PATS and AEC1 marker genes. Open box highlights bleomycin-specific cluster 10 with shared PATS and AEC2 markers (“Fibro-PATS”). (**F**) RNA velocity plot: open box highlights clusters 8 (AEC1), 10 (Fibro-PATS), and 3 (PATS).

The condensed, spherical morphology and increased HOPX expression observed in organoids from bleomycin-derived FBs provided an intriguing insight into the effect of fibrogenic *Pdgfra^+^* FBs on AEC2 homeostasis and response to injury. To investigate this phenomenon at the transcriptomic level, we conducted scRNA-Seq on alveolar epithelia from organoid cultures grown with normal or fibrogenic FBs, harvested after 2 weeks in culture (Figure 9A). Analysis of the epithelial population revealed 14 independent clusters (Figure 10A and Supplemental Table 9; quality control data in Supplemental Table 1). The distribution and percentage cell proportions of PBS- and bleomycin-derived epithelial cells are shown in Figure 10, B and C, respectively. We first confirmed the presence of key cell populations that should be extant in fully mature organoid cultures. Those include AEC2 (*Sftpc^+^*), AEC1 (*Hopx^+^*), proliferating alveolar cells (*Top2a^+^*), and the recently described pre-alveolar type-1 transitional state (PATS) (36) population, shown in Figure 10D. Genes previously identified as characteristic of PATS or AEC1 (36) were found in cluster 10, which is largely derived from fibrogenic FB organoids (Figure 10, B and C), suggesting that cluster 10 is an intermediate transitional population unique to the presence of profibrotic FBs (“Fibro-PATS,” Figure 10E). RNA velocity analysis (Figure 10F) showed that cluster 8 (AEC1) is the terminal cluster into which cluster 3 (PATS) flows, transitioning through cluster 10 (Fibro-PAT) (clusters highlighted in open box) and establishes a transcriptional relationship between these populations, similar to what was shown by Kobayashi et al. (36). Additionally, clusters involving AEC2 flow to AEC1 as the terminus, further establishing these cells as the differentiation endpoint. Cluster 12, which is derived from normal FB–supported organoids (Figure 10, B and C), is made up of 2 distinct subclusters, 12a and 12b (Supplemental Figure 14, A and B). We found that subcluster 12b is *Sftpc*^–^ and PATS^+^ and has gene markers in common with the PATS signature (Supplemental Figure 14C), while subcluster 12a is *Sftpc*^+^ and PATS^–^ but is uniquely enriched in mitochondria-encoded genes (Supplemental Figure 14C) and RNA velocity analysis connects it to the PATS population (Figure 10F), thus supporting the transitional status of PATS between AEC2 and AEC1.

Finally, we were interested in assessing the transitional populations we describe here in the context of p53 signaling and senescence, which are characteristic of PATS (36). We found that while the Fibro-PATS population shared some genes in common with PATS for both senescence and *p53* signaling (Supplemental Figure 15), the PATS^+^ population found in normal FB–supported organoids did not express those genes (Supplemental Figure 15). Taken together, these data demonstrate that normal and fibrogenic *Pdgfra*^+^ FBs have distinct and unique effects on AEC2 progenitor differentiation and metabolic requirements, as well as the evolution of AEC2 transitional populations.

## Discussion

Epithelial repair and regeneration of the alveolar stem cell niche is critical for restoration of normal lung function following injury. Dysregulation of these processes underlies pathological disease progression in IPF, where chronic injury to alveolar epithelia can over time overwhelm the organized regeneration of the alveolus, leading to loss of epithelial integrity. AEC2 progenitors and resident *Pdgfra*-expressing LipoFBs that form the alveolar niche function coordinately in support of progenitor homeostasis and response to injury, and disruption of this axis leads to disease progression in fibrosis. Several theoretical models of IPF pathogenesis support a so-called multiple-hit hypothesis, whereby repeated insults render normal alveolar repair mechanisms deficient (37). In our studies, we questioned whether an initial injury to the LipoFBs may affect their ability to support alveolar regeneration and to that end, determined transcriptomic and functional changes that occur in *Pdgfra*^+^ FBs in bleomycin-injured mouse lung.

We focused on the identification and characterization of the *Pdgfra^+^* LipoFB subpopulation in normal and fibrotic lung, as this population along with AEC2s forms the alveolar epithelial stem cell niche (9, 38), supports AEC2 progenitor function and regeneration (39, 40), but also potentially provides a MyoFB precursor pool upon injury (13). LipoFBs are found at the base of the developing alveolar septa (41) in close proximity to AEC2s (6, 9, 11). The presence of LipoFBs in human lung has been more difficult to establish, but recent evidence supports their presence in normal (42) and fibrotic human lungs (43). Thus, it is important to understand how lung injury affects the structure and function of these cells. Our data support 2 major conclusions: first, fibrotic lung injury leads to a fundamental reprogramming of resident LipoFBs; second, these LipoFBs have an impaired ability to support epithelial regeneration and differentiation.

We found that LipoFBs derived from bleomycin-injured lung represent a transitional state between normal LipoFBs and MyoFBs. Using a nonsupervised analysis of the 11 unique clusters in our integrated data set, we identified a shared transcriptomic signature between normal and injured LipoFBs, consisting of 22 genes that annotated to both lipogenic and fibroblastic functions, both which are defining features of this population. LipoFBs play an integral role in supporting AEC2 function by providing lipid substrate for surfactant production; however, the neutral lipid contained in LipoFBs also serves an antioxidant role, protecting alveolar epithelial cells from oxygen free radicals (44). Therefore, mechanisms that preserve the lipogenic FB phenotype in both naive and injured lung are critical. For example, we identified carboxylesterase 1D (*Ces1d*) as one of our conserved LipoFB genes. Broadly, carboxylesterases have roles in the metabolism of toxicants and xenobiotics (45), which serve to preserve lipid integrity, and CES1D has been shown to mediate lipid droplet homeostasis (46, 47). Protection against oxidative stress is important in preserving lipid integrity, and the antioxidant enzyme superoxide dismutase 3 (*Sod3*) is another key gene in the shared lipogenic profile between normal and injured cells. Superoxide dismutases are important antioxidants (48), and in addition, SOD3 binds to ECM components (49, 50), and has an important protective role in hyperoxia-mediated alveolar injury (51).

LipoFBs are also characterized by the presence of contractile filaments (reviewed in refs. 10, 43). Genes that annotate broadly to ECM-related functions in our data set, including *Npnt* (ECM organization and adhesion), *Cola13a1* and *Col23a1* (cell-matrix and cell-cell adhesion), and *Macf1* and *Shroom3* (actin filament interactions) support a functional mesenchymal cell phenotype. *Npnt* and *Macf1* were found to be LipoFB-specific markers (17), and *Col13a1* is associated with the LipoFB gene signature (16, 17). In summary, we describe a minimal set of genes that may be collectively important for maintaining the phenotypic and functional integrity of LipoFBs in normal and in injured tissue.

Using an antibody against PLIN2 (ADRP), one of the conserved LipoFB genes in our list that is a canonical LipoFB marker and is functionally involved in the formation of lipid droplets (52), we demonstrated LipoFB localization in preserved alveoli in fibrotic mouse lung (Figure 2B). However, while they retain sufficient identity as LipoFBs, in terms of their location and expression of a fundamental LipoFB gene panel, injured LipoFBs do suffer a loss of lipogenic support pathways in tandem with activation of fibrogenic pathways, as shown in our comparative gene expression profile (Figure 4). LipoFBs are uniquely spatially situated to support AEC2 function by providing lipids for surfactant production (53) as well as having an antioxidant role (54). In contrast, MyoFBs are unable to support stem cell proliferation (40). Thus, LipoFBs that have acquired a MyoFB phenotype, even partially, may not support alveolar epithelial cell function as effectively.

**Figure 11:**
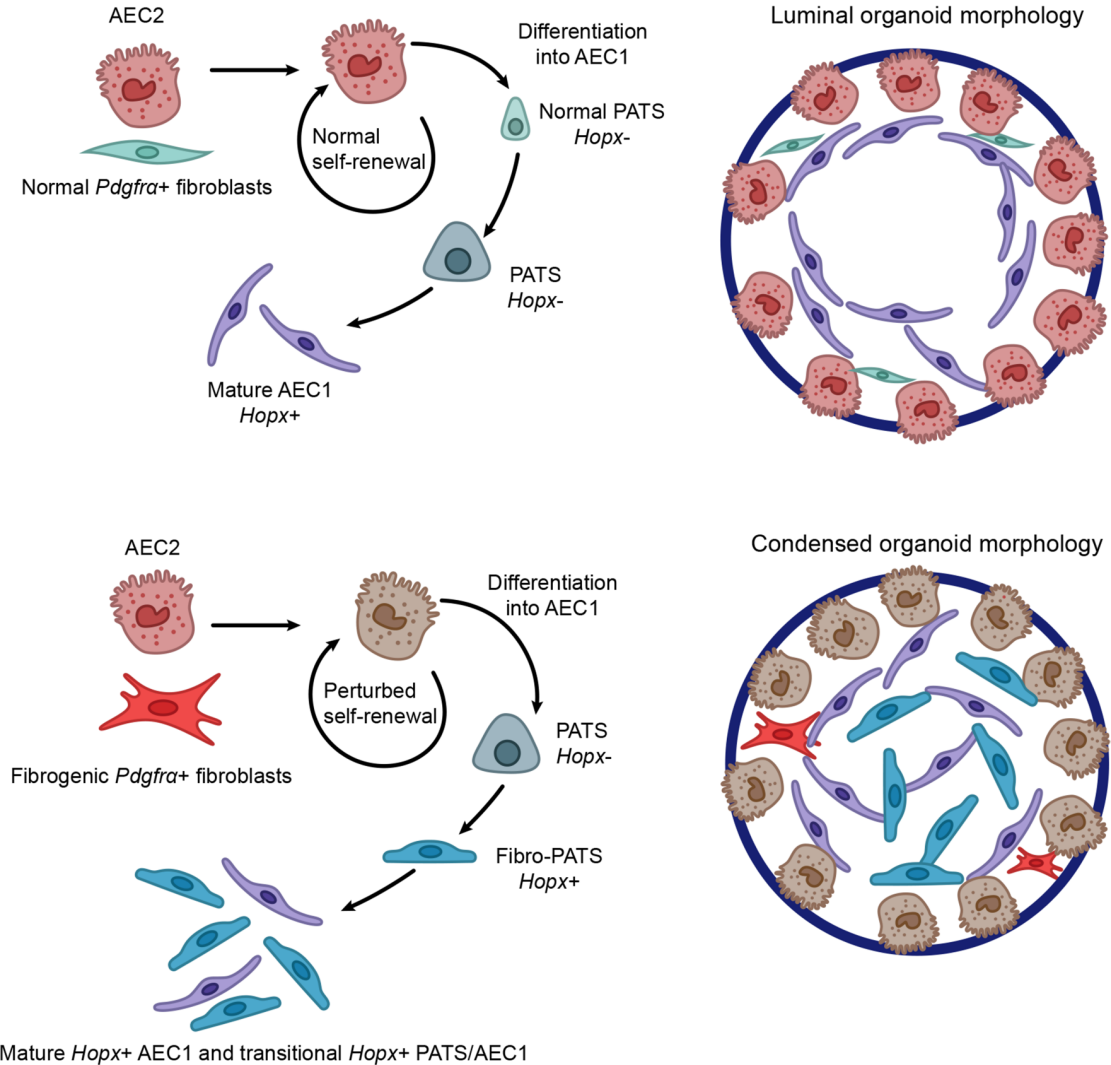
Graphical illustration of the effect of normal and fibrogenic *Pdgfra*^+^ fibroblasts on AEC2 self-renewal and differentiation. Top: AEC2s combined with control *Pdgfra*^+^ fibroblasts result in normal AEC2 self-renewal and AEC1 differentiation. Normal PATS (*Hopx*^–^) are positioned early in the PATS transitional pathway, and luminal organoids develop. Bottom: AEC2s combined with fibrogenic *Pdgfra*^+^ fibroblasts undergo perturbed self-renewal. Fibro-PATS occur in later stages of the transitional PATS pathway, resulting in a combination of mature AEC1 (*Hopx*^+^) and hybrid PATS-AEC1 (*Hopx*^+^) that form in the densely compacted organoid interior.

To test this hypothesis, we used the well-established method of lung organoid culture, where AEC2 progenitors are cocultured with lung *Pdgfra^+^* FBs that are necessary for AEC2 differentiation and the formation of self-organized alveolospheres (9). We found that *Pdgfra^+^* FBs from bleomycin-injured lungs could support AEC2 differentiation, but the morphology of the developed organoids was less structured, in that there was an absence of lumen formation as would have been expected in a lung organoid. Instead, organoids arising from fibrogenic *Pdgfra^+^* FBs had a densely compacted organization of HOPX^+^ AEC1s within the center of the organoid, with little evidence of the typical long extensions between cells seen in alveolospheres (9), as well as a trend for HOPX^+^ cells to occupy a greater percentage of the total area of the condensed organoids in bleomycin versus PBS cultures. Using scRNA-Seq analysis of the alveolar epithelia from these organoid cultures, we demonstrated distinct transcriptomic changes in these cells, with several cell clusters arising predominantly or exclusively from the normal or fibrogenic FB–supported populations, respectively. Notably, there appeared to be a significant shift in the behavior of transitional PATS cells. Although both conditions shared a common PATS population, additional PATS cells (cluster 12b, Supplemental Figure 14B) from normal FB organoids lacked both AEC2 and AEC1 markers and senescence/*p53* pathway–related genes, while the unique Fibro-PATS from the fibrogenic FB cultures (cluster 10, Figure 10E) carried a distinct AEC1 signature. Thus, it is possible that the normal PATS population is overall positioned at an earlier point in the transitional trajectory, where they have lost AEC2 identity but have not yet acquired the full PATS profile. On the other hand, Fibro-PATS are found, perhaps stuck in transition, at a later transitional point and may contribute to the increased number of HOPX^+^ cells observed in the condensed organoids derived from fibrogenic FBs. This is summarized in the graphical illustration in Figure 11.

Our data are supported by published in vivo evidence; for example, following bleomycin exposure, AEC2 differentiation into AEC1 is increased in the mouse lung (55, 56). TGF-β1 has been shown to mediate AEC2 differentiation into AEC1, acting either through the SMAD pathway (57) or via an inverse relationship with BMP signaling (58). Our cluster analysis demonstrates that the activated MyoFB component of the fibrogenic *Pdgfra^+^* population as well as injured LipoFBs exhibit upregulated *Tgfb1* expression, while there is little expression in normal LipoFBs, providing a source for paracrine regulation of AEC2 differentiation in culture supported by injured FBs. Therefore, increased *Tgfb1* expression derived from injured *Pdgfra*^+^ FBs may be one underlying factor mediating the difference in alveolosphere morphology. Indeed, it was recently shown that the addition of a profibrotic cocktail to alveolosphere culture induces a similar condensed organoid morphology as well as the appearance of transitional populations (59), supporting the conclusion that interstitial lung FBs can directly affect alveolar epithelial cell functional responses.

Condensed organoids were also seen in a study using aged FBs as support cells (60). IPF is a disease of the aged lung, and FBs in IPF lungs have a senescent phenotype that has been shown to contribute to the development of fibrosis (61–64). Our data support the notion that lung injury may move LipoFBs to behave in similar ways to aged LipoFBs and thus alter their ability to support regeneration of the lung in response to subsequent injury. For example, the gene *Ppp1r15a*, which was recently shown to suppress senescence and fibrosis (65), was notably downregulated in our fibrotic LipoFBs, while we saw an upregulation of the senescence-associated gene *Serpine2* (Supplemental Figure 16) (65). Previous research has shown that aged or young FBs are able to determine the fate of alveolar cells in organoid culture (34). Our study expands on these findings by showing that injured LipoFBs possess similar properties and suggests that LipoFB injury is an important contributor to aberrant lung function in fibrosis.

In addition to pathway modeling, our pseudotime analysis offers further insight into how LipoFBs respond to injury. Normal LipoFBs can progress through a loop trajectory, where they transit through injured LipoFBs and then back to normal through acquisition of fibrogenesis-related genes and retention of lipogenesis-associated genes (Figure 4, A and D). This combination suggests an injury-activated wound healing response, supporting regeneration of the matrix. On the other hand, normal LipoFBs that transit to MyoFBs in the linear trajectory do so through the injury-activated LipoFB-related cluster 7, which, while having similar levels of fibrogenic genes, has a significant reduction in lipogenic gene expression compared with the canonical injured LipoFBs in cluster 6 (Figure 4, C and D). Indeed, the genes that form the common LipoFB signature are absent from cluster 7 (Figure 3, A and B). Therefore, these data suggest that progressive attenuation of lipogenic gene expression is a primary driver of injured LipoFBs acquiring a pathologic rather than a regenerative phenotype. Thus, we postulate that our alveolosphere results, showing a shift in epithelial phenotype and transcriptional signature when AEC2s are cocultured with *Pdgfra^+^* FBs from fibrotic lungs, was driven by a shift toward a more pathologic LipoFB phenotype.

Our results have relevance to human IPF and fibrosis-induced decline in lung function. Using a combination of data mining and multiomics analyses we demonstrate that our gene signature in FBs, obtained on day 14 after a single bleomycin exposure, represents the peak fibrotic transcriptional perturbation of this model in the bulk lung transcriptome. The gene signatures of MyoFBs are strongly correlated with alteration in lung function and are coexpressed with previously described gene coexpression networks G-1 and G-2, which are the major drivers of progressive fibroproliferative changes in the lung transcriptome (33). Furthermore, they associated with gene expression signatures in IPF lungs, as well as lung function in IPF patients. Thus, our results validate the use of this model and time point as a tool to, for example, study therapeutic interventions in a targeted, parsimonious way.

In conclusion, our results suggest that LipoFB populations respond to fibrotic lung injury by altering their gene expression profile, losing lipogenic markers and gaining profibrotic markers, which in turn impairs their ability to support stem cell niche growth and development. Our research may help explain how injury promotes progressive fibrosis by impacting the ability of the stem cell niche to respond to subsequent triggers and suggests that drugs targeting this pathway may allow for reversibility of the process to improve in lung function and disease outcomes.

## Methods

### Animals.

*Pdgfra^GFP^* mice (B6.129S4-*Pdgfra^tm11(EGFP)Sor^*/J, stock number 007669) were obtained from The Jackson Laboratory (66). Male mice were used in these studies due to enhanced sensitivity to bleomycin-induced fibrosis compared with females (67, 68), and single-housed mice were 10–14 weeks of age when used. Tamoxifen-inducible *SpcCreER* × *Rosa-td26* mice were generated for isolation of tomato-expressing SFTPC-positive AEC2s by crossing B6.Cg-GT(ROSA)*26Sor^tm9(CAG-tdTomato)Hze^*/J (The Jackson Laboratory, stock number 007914) with B6.129S-*Sftpc^tm1(creERT2)Blh^*/J (The Jackson Laboratory, stock number 028054).

### Induction of PF.

*Pdgfra^GFP^* reporter mice were placed under oxygen/isoflurane anesthesia and dosed with 2 U/kg bleomycin (Sigma-Aldrich) or 1× PBS in a 50 μL volume by oropharyngeal aspiration. Lungs were collected on day 14 after exposure for preparation of live cells for scRNA-Seq, fixation for histology, immunofluorescent staining, or 3D organoid (alveolosphere) culture. Details of tissue dissociation and live cell isolation are provided in Supplemental Methods.

### scRNA-Seq.

*GFP^+^* cell suspensions prepared from PBS- and bleomycin-treated *Pdgfra^GFP^* mice (described above) were counted and examined for viability using a TC-20 cell counter (Bio-Rad). Approximately 1,800 live cells at 1 × 10^6^ cells/mL concentration with greater than 80% viability were loaded into the Single Cell Chip followed by forming single cell emulsion in Chromium Controller (10× Genomics, Chromium Single Cell 3′ Library & Gel Bead Kit v2). The mRNA reverse transcription and cDNA generation and amplification were carried out followed by the construction of single-cell gene expression libraries according to the protocols provided by the manufacturers. The libraries were then sequenced by the National Institute of Environmental Health Sciences (NIEHS) Epigenomics and DNA Sequencing Core Laboratory on a NextSeq 500 (Illumina) with paired-end sequencing (read 1: 30; read 2: 100). A total of 3.1 × 10^8^ reads were obtained.

### scRNA-Seq data analysis.

Sample fastq files were processed with Cell Ranger v3.0.1 (10× Genomics) using the GRCm38 genome and Gencode version M17 (https://www.gencodegenes.org/mouse/release_M17.html; downloaded March 22, 2018 and filtered according to 10× Genomics recommendations) to generate an initial cell-by–gene count matrix. All samples were combined in Seurat v3 (69) and clustered following SCTransform (70) normalization. In each cluster, cells were removed if their mean absolute deviation (MAD) for percentage of mitochondrial genes or erythrocyte markers was greater than 3. Additionally, any cluster with a mean expression of mitochondrial genes greater than 25% or erythrocyte markers greater than 0.5% was removed. Finally, known markers were used to remove small contaminating clusters of epithelial cells, endothelial cells, macrophages, T cells, and B cells, leaving only FBs for downstream analysis. Clustering of the PBS- and bleomycin-exposed lung cells together produced 11 clusters, with only 2 showing significant overlap between the samples. Markers for these genes were found using MAST (71) considering percentage mitochondrial genes as a latent variable. Slingshot (72) was run using the Seurat-generated UMAP as the reduced dimension. RNA velocity estimations were computed using the velocyto package (73) and visualizations were created by scVelo (74).

### Tissue preparation for immunofluorescent staining.

Lungs were first perfused with 1× PBS, and then inflated with either 10% neutral buffered formalin or 4% paraformaldehyde (in PBS). Formalin-fixed tissues were paraffin embedded following routine histological processing and 5-μm sections prepared for staining. Paraformaldehyde-fixed tissues were washed in 1× PBS, placed into 30% sucrose (in PBS) overnight at 4°C, and then changed to OCT/30% sucrose (1:1) and left at 4°C overnight. Lungs were placed into cryomolds on dry ice and frozen in OCT and sectioned at 5 μm. Extended methods for immunofluorescent staining are available in Supplemental Methods. Table 1 lists all primary and secondary antibodies.

### scRNA-Seq data mining.

scRNA-Seq data sets were obtained from the NCBI Gene Expression Omnibus (GEO) database and analyzed using the Seurat v4.2.0 R package (https://satijalab.org/seurat/). Only control samples, bleomycin-induced data, or IPF patient data were selected for further analysis. Low-quality cells were filtered out at both cell and gene levels. Cells were removed if they had greater than 10% unique molecular identifiers (UMIs) derived from the mitochondrial genome, or if their total UMIs or genes fell outside the upper and lower bounds (defined as mean ± 2 SD). Genes with zero counts or those expressed in 10 or fewer cells were also excluded. The remaining cells were normalized using Seurat’s SCTransform function, and highly variable genes were identified using the FindVariableFeatures function. The data were then scaled with Seurat’s ScaleData function. The number of principal components (PCs) for clustering was determined using an elbow plot, and the resolution for clustering was determined using clustree R package v0.5.0 (https://github.com/lazappi/clustree). FB types were defined using positive (*Pdgfra*, *Col1a1*, and *Acta2*) and negative (*Epcam* and *Pecam1*) canonical markers. FBs were extracted from the whole-lung single-cell data set based on cell barcodes. The FB subset data were then reanalyzed, clustered, and annotated using the top markers from Figure 1D.

### Analysis of FB marker genes in PF and lung function.

To further understand the role of the FB marker genes in the progression of IPF and their association with pulmonary functions, we retrieved the pulmonary functions, lung transcriptomics data, and gene coexpression network from the Mouse Lung Fibrosis Atlas (https://niaaa.nih.gov/MouseLungFibrosisAtlas) (33) and filtered them based on the gene cell marker data from the scRNA-Seq analysis results. Transcriptomics expression data were reexpressed as normalized counts, specifically *Z*-score normalization of the transcript per kilobase million (TPM) values and, for the network visualization, only pulmonary functions and selected gene cell markers are shown. The gene cell markers were selected based on whether they were significantly altered 14 days after bleomycin in bulk lung transcriptomics data (from the Mouse Lung Fibrosis Atlas) and met marker selection criteria (FDR-adjusted *P* value < 0.01 and fold change > 0) for the indicated cell group.

### Protein isolation and analysis.

*Pdgfra^GFP^* reporter mice were dosed with either PBS or bleomycin as described above. GFP*^+^* cells were isolated by FACS from 4 biological replicates, combining lung digests from 3 PBS and 3 bleomycin-dosed mice per replicate. Extended methods can be found in Supplemental Methods.

### Proteomics and multiomics.

Protein normalized abundance measurements were log_2_-transformed and median-normalized. A total of 2,777 proteins were identified that met the criteria of presence in at least 2 samples in at least 1 group (PBS or bleomycin). Statistical analysis employed the limma v3.42.2 package (https://bioconductor.org/packages/release/bioc/html/limma.html), with a post hoc adjustment performed by the DEqMS v1.4.0 package (https://www.bioconductor.org/packages/release/bioc/html/DEqMS.html) designed for MS proteomics data that accounts for the number of peptide spectral matches for each protein. Statistical hits were defined as bleomycin samples having an adjusted *P* value of less than 0.05 and an absolute fold change of 2.0 or greater relative to control PBS samples. Three hundred ninety-four statistical hits (179 up, 215 down) were identified. Heatmaps were produced using the ComplexHeatmap v2.15.1 package (75).

### 3D organoid culture.

Organoid cultures were prepared as described in Barkauskas et al. (9). Sorted GFP*^+^* FBs from PBS- (*n* = 3) or bleomycin-exposed (*n* = 3) mice were combined with SFTPC-tomato^+^ AEC2s (5 × 10^5^ and 5 × 10^3^, respectively) with growth factor–reduced Matrigel (Corning; 1:1 cells/Matrigel). Of this, 90 μL was pipetted into 24-well 0.4-mm PET Transwell inserts (Falcon) and allowed to polymerize for 30 minutes in a 37°C/5% CO_2_ incubator. After polymerization, 600 μL of MTEC/Plus media (9) was added to each well. At plating, media for the cell/Matrigel mixture and media for culture were supplemented with ROCK inhibitor (10 μM; Sigma-Aldrich). After the initial 48 hours, culture media were refreshed every other day for 14 days without ROCK inhibitor supplementation. On day 14, live cultures were imaged with a Zeiss automated inverted epifluorescence microscope at ×5 magnification and images captured with Zen Blue software (Carl Zeiss, Inc). Matrigel inserts were then fixed in 10% neutral buffered formalin overnight at room temperature, washed in PBS, and stored at 4°C prior to whole-mount staining. Details for whole-mount staining, organoid measurement, and dissociation for scRNA-Seq are available in Supplemental Methods.

### Statistics.

Graphs and statistical analysis were accomplished with GraphPad Prism, v9.3.0. Data are presented as mean ± SD, and statistical comparisons were made with the unpaired, 2-tailed Student’s *t* test and 2-way ANOVA, or in Proteome Discoverer, using 2-way ANOVA. Differences were considered significant if *P* was 0.05 or less.

### Study approval.

All animals were housed in the NIEHS animal facility, provided food and water ad libitum, and all procedures were conducted under approved NIEHS Institutional Animal Care and Use protocols.

### Data availability.

The scRNA-Seq data are available in the NCBI GEO, accession number GSE183423. Raw data files for graphs in Figure 9, C, D, and G, and Supplemental Figure 1C are available in the Supporting Data Values file. All data are available from the corresponding author upon request.

## Author contributions

CST, AKP, and SG conceived the experiments and wrote the manuscript. CST performed experiments and analysis. BNP contributed biostatistical analysis. XX, MIS, and CDB contributed flow cytometry and sorting strategies and experiments. ES and CJT contributed imaging data and analysis. KLJ, LJD, JGW, and DJJ contributed protein isolation and LC-MS/MS, proteomics, and multiomics analyses. JLL performed data mining experiments. MA and RC contributed analysis of scRNA-Seq data with the fibrosis and lung physiology database. AB and LP performed in vivo experiments. DS, CG, and DM contributed TEM processing, imaging, and analysis.

## Supplementary Material

Supplemental data

Supplemental table 2

Supporting data values

## Figures and Tables

**Table 1:**
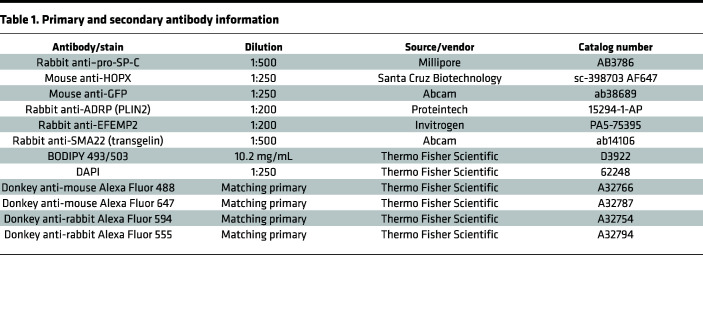
Primary and secondary antibody information

## Version 1 10/12/2023

In-Press Preview

## Version 2 11/22/2023

Electronic publication
