# Supplementary material for: Functional Pdgfra fibroblast heterogeneity in normal and fibrotic mouse lung
Source: JCI Insight. 2023 Nov 22;8(22):e164380. doi: 10.1172/jci.insight.164380 (PMC10721331; doi:10.1172/jci.insight.164380)
Supplement: Supplemental data [file jciinsight-8-164380-s056.pdf]

Supplemental Materials and Methods.

Immunofluorescence staining. Formalin-fixed, paraffin embedded (FFPE) sections were deparaffinized in Xylenes and rehydrated to 1XTBST through graded ethanol. Frozen sections were air dried and rehydrated in 1XTBST. FFPE sections were incubated 10 minutes in 10% aqueous hydrogen peroxide, washed in 1XTBST, then subjected to antigen retrieval in either 1X decloaking solution (BioCare Medical) or 1XEDTA antigen retrieval solution (Fisher Scientific, Houston, TX) using a pressure cooker (BioCare Medical, Pacheco, CA). Frozen tissues were first permeabilized in 0.5% TBST. Tissues were blocked in 10% normal donkey serum (Jackson ImmunoResearch, Baltimore Pike, West Grove, PA), 1% BSA, in 1XTBST, and incubated with primary antibodies (rabbit anti-ADRP (PLIN2), 1:200; rabbit anti-EFEMP2 1:200; mouse anti-GFP, 1:250; rabbit anti-SMA22 1:500) or serum controls overnight at 4°C. Secondary antibodies were donkey anti-rabbit or donkey anti-mouse AlexaFluor conjugates. Antibody information is available in Table 1. Slides were incubated 1 hour at room temperature, washed, and then stained with DAPI (300 nM in PBS; Fisher) for 15 minutes at RT. In experiments where BODIPY (Fisher) was used, 2.65 ml of a 1 mg/ml stock in DMSO was added in with the secondary antibodies. Cover slips were affixed with Prolong Gold Diamond (Fisher Scientific) and staining was assessed with an Olympus BX-51 microscope (Olympus Scientific Solutions Americas, Waltham, MA) fitted with an Olympus DP-80 digital camera software (cellSens Standard 1.18), or with a Zeiss LSM 880 inverted confocal microscope with AiryScan (Carl Zeiss, Inc, Oberkochen, Germany) and Zeiss Zen software.

Tissue dissociation and live cell isolation by FACS. Lungs were collected from three PBS-treated (normal) or three bleomycin-treated (fibrotic) *Pdgfra-GFP* reporter mice 14 days post exposure and dissociated as previously described (Barkauskas CE, et al. 2013). *SpcCreER X Rosa-td26* mice were dosed 2-3 times with Tamoxifen (Sigma Aldrich, St. Louis, MO; 0.2 mg/10g body weight IP in corn oil) IP and lungs from two mice were harvested one week after the last Tamoxifen dose

for experimental use. Briefly, following euthanasia, lungs were perfused with cold 1XPBS, and inflated with dissociation solution (Collagenase Type 1 (450 U/ml, Invitrogen), Elastase (4 U/ml; Worthington), Dispase (5 U/ml; Fisher Scientific), and DNase1 (0.33 mg/ml; Sigma) in DMEM/F12 media). Lungs were minced with scissors, then added to 3 ml of dissociation solution, one lung per tube in 15 ml centrifuge tubes. Lungs were digested at 37°C for 25-30 minutes, quenched with DMEM/F12 plus 10% FBS, and dissociated tissues from the individual tubes were combined (3 PBS and 3 Bleomycin) then filtered through 100-micron cell strainers. Filtered cells were pelleted, red blood cells lysed with 1XRBC lysis buffer (Fisher Scientific), then filtered through 40-micron cell strainers. After washing once in DMEM/F12, cells were resuspended in buffer (2% BSA in DMEM/F12), filtered through 30-micron cell strainers, and GFP+ cells were collected using a BD FACSAria II (BD Biosciences). Dead cells were gated out using Propidium Iodide (20 ml/ml of a 1 mg/ml stock). Sorted *Pdgfrα*^{GFP} positive cells from both normal and fibrotic lungs contained both GFP^{bri} and GFP^{dim} populations, (Green et al. 2016), although the GFP^{bri} population was attenuated in fibrotic lung (Supplemental Figure 1A and B, PBS and Bleomycin, respectively), referred to hereafter as GFP+. The percent single cells from FACS analysis was determined from 7 independent experiments, demonstrating a significant reduction (p=0.0011) of GFP+ cells from bleomycin-treated mice compared to PBS controls (Supplemental Figure 1C). Tomato+ AECII cells from tamoxifen-induced *SpcCreER X Rosa-td26* mice were sorted from lung homogenates for use in 3D organoid cultures.

Ingenuity Pathway Analysis of canonical pathways. Individual cluster data was uploaded into Ingenuity Pathway Analysis (IPA version 01-20-04) for canonical pathway analysis (pathway data are available in Supplemental Tables 3, 4, and 5 for clusters 0, 6, and 9, respectively). For the pathways of interest, the percent downregulated and upregulated genes annotated to each pathway were put into GraphPad Prism (v9.3.0) to construct bar charts.

Brightfield images of Masson's Trichrome stained normal and fibrotic mouse lung. Lungs from bleomycin or PBS-exposed mice (Figure 1A) were fixed with 10% Neutral Buffered Formalin (NBF) and processed through routine histological procedures to generate formalin-fixed, paraffin embedded sections (FFPE). Sections were stained with Masson's Trichrome (NIEHS Histology Core) and imaged using Aperio Imagescope. Slides were first cleaned with isopropanol, then scanned using the Aperio AT2 Scanner (Aperio: Leica Biosystems Inc., Buffalo Grove, IL) line scanning technology to capture high resolution, seamless digital images of glass slides. Slides were viewed using the digital slide viewing program WebViewer (Aperio), which was used to capture images.

Repetitive Bleomycin exposure, tissue dissociation, and cell isolation by FACS. *Pdgfrα*^{GFP} mice reporter mice were dosed with either Bleomycin or PBS once weekly for a total of three weeks. Bleomycin consecutive doses were 1 U/Kg, 1 U/Kg, and 0.5 U/Kg in 50 ul PBS administered by oropharyngeal instillation under isofluorane/oxygen anesthesia. Control mice were given PBS in a 50 ul dosing volume. Two weeks after the last of 3 doses, lungs were collected, enzymatically digested, and three lungs per treatment group were pooled for collection of GFP+ fibroblasts by FACS.

Single Cell RNA sequencing repetitive bleomycin exposure. Gene expression data is available in Gene Expression Omnibus (GEO), accession number GSE183423. Quality control information is in Supplemental Table 1.

Protein isolation. Cells were lysed and protein extract generated using Thermo Fisher EasyPep Mini MS sample preparation kit. Protein content was quantified using the Bradford Assay, then proteins were reduced, carbamidomethylated, and digested with Lys-C and trypsin. After peptides

were purified using spin columns, each of the 8 samples were analyzed in triplicate using LC-MS/MS. Protein digests were analyzed by LC-MS/MS on a Q Exactive Plus mass spectrometer (ThermoFisher Scientific) interfaced with a nanoAcquity UPLC system (Waters Corporation) equipped with a 75 □m x 200 mm HSS T3 C18 column (1.8 □m particle, Waters Corporation) and a Symmetry C18 trapping column (180 □m × 20 mm) with 5 □m particle size at a flow rate of 450 nL/min. The trapping column was positioned in-line of the analytical column and upstream of a micro-tee union which was used both as a vent for trapping and as a liquid junction. Trapping was performed using the initial solvent composition. Approximately 1 mg of peptide digest was injected onto the column. Peptides were eluted by using a linear gradient from 99% solvent A (0.1% formic acid in water (v/v)) and 1% solvent B (0.1% formic acid in acetonitrile (v/v)) to 40% solvent B over 100 minutes. For the mass spectrometry a data dependent acquisition method was employed with a dynamic exclusion time of 15 seconds and also exclusion of singly charged ions. The mass spectrometer was equipped with a NanoFlex source and a stainless-steel needle and was used in the positive ion mode. Instrument parameters were as follows: sheath gas, 0; auxiliary gas, 0; sweep gas, 0; spray voltage, 2.7 kV; capillary temperature, 275 °C; S-lens, 60; scan range (m/z) of 375 to 1500; 1.6 m/z isolation window; resolution: 70,000; automated gain control (AGC), 3 × 10e6 ions; and a maximum IT of 100 ms. For the MS/MS scans: TopN: 10; resolution: 17500; AGC 5 x10e4; maximum IT of 50 ms; and an (N)CE: 27. Mass calibration was performed before data acquisition using the Pierce LTQ Velos Positive Ion Calibration mixture (ThermoFisher Scientific). Data were processed using Proteome Discover also from ThermoFisher (https://www.thermofisher.com/) using a processing workflow that employed nodes for the Minora Feature Detector, Sequest HT, and Percolator. Data were searched against the mouse uniprot sequence database using Sequest and included settings of 1) trypsin specificity; 2) allowance for two missed cleavages; 3) 20 ppm mass tolerance for MS; 4) 0.6 Da mass tolerance for MS/MS; 5) static modification of cysteine residues with carbamidomethylation; 6) variable methionine oxidation; and 7) variable modification of asparagine and glutamine

deamidation. A consensus workflow containing Feature Mapper and Precursor Ion Quantifier allowed for label-free quantification.

Pathway enrichment analysis. Protein identifiers were updated using Bioconductor to current Entrez gene symbols for pathway enrichment analysis and comparison with transcriptomic data. The msigdbr-7.5.1 package was used to generate a canonical pathway gene set and statistical protein hits were tested for enrichment using Gene Set Enrichment Analysis (GSEA) via the clusterProfiler-3.14.3 package, using a false discovery rate (FDR) adjusted P-value <0.05 and at least four significantly regulated proteins. For the upregulated and downregulated proteins, a protein-pathway incidence matrix was created using the top 15 canonical pathways and the corresponding genes for each pathway.

Organoid measurement. After 14 days in culture, epifluorescence images were captured of each Matrigel culture (Figure 4B shows representative images, all images (9 each per group) are shown in Supplemental Figure 5A and B, PBS and Bleomycin derived cultures, respectively) to assess colony number and size using tomato+ fluorescence. Epifluorescence images were taken on a Zeiss AxioObserver.Z1 microscope (Carl Zeiss Inc, Oberkochen, Germany) using a HXP 120 C metal halide light source for excitation coupled with an AxioCam Mrm camera and a Fluor 5X/0.25 M27 objective. Fluorescence excitation/emission of the red channel was achieved using Zeiss filterset #43. The raw CZI images were opened in ImageJ version 1.53n and a macro was written to perform background subtraction, smoothing, and apply a threshold to create a binary image. Watershed was applied to segment the binary image and then Analyze Particles was used to perform a size filter and then measure the Feret Diameter of each organoid.

Immunofluorescence staining of 3D organoid cultures. Whole mount staining was conducted based on a protocol provided by the laboratory of Dr. Carla Kim (Harvard Stem Cell Institute;

personal communication). In brief, fixed cultures (retained on the Transwell mesh) were permeabilized in 0.5% TBST for 1 hour at RT, then blocked overnight in 0.2% TBST supplemented with 10% normal donkey serum. Purified rabbit anti-pro-SP-C (SFTPC; 1:500; Millipore) was added to inserts in 0.2% TBST and incubated overnight at 4°C. The next day, inserts were washed in 0.2% TBST, secondary antibody for Spc (Donkey anti-rabbit Alexa Fluor 488, 1:500; Invitrogen), mouse anti-HOPX-Alexa Fluor 647 (1:250; Santa Cruz Biotechnology, Inc, Dallas, TX), and DAPI (1:250; Fisher Scientific) were added to inserts in blocking diluent followed by an overnight incubation at 4°C. Whole mounts were imaged with a Zeiss LSM 880 inverted confocal microscope with AiryScan (Carl Zeiss, Inc, Oberkochen, Germany) fitted with a Plan-Apochromat 20x/0.8 M27 objective, and Zeiss Zen software.

Dissociation of organoids and Single Cell RNA Sequencing. Organoid cultures in the Transwell inserts were digested in 1X Accutase (Thermo Fisher) for 10 minutes at 37°C/5% CO_2. Using a wide-bore pipette tip, organoids were removed to 15 ml centrifuge tubes and digested at 37°C in Accutase for an additional 10 minutes with frequent mixing. Cells were pelleted, then digested in 0.25% Trypsin-EDTA at 37°C for 10 minutes with frequent mixing. After pelleting, cells were resuspended in media, counted, and prepared for scRNA-Seq and analysis as described above.

Transmission Electron Microscopy of 3D organoid cultures. Following 2 weeks of culture, transwell inserts with attached organoids were fixed in Trump's fixative (4% formaldehyde:1% Gluteraldehyde). Samples were rinsed with buffer, post-fixed in 1% osmium tetroxide in phosphate buffer, rinsed with distilled water, and dehydrated in an ethanol series transitioning to a 1:1 solution of ethanol and Poly/Bed 812 epoxide resin (Dodecenylsuccinic Anhydrid, Nadic Methyl Anhydride, DMP-30, all from Polysciences, Inc, Warrington PA). Fixed inserts were left overnight at room temperature to allow infiltration of the Poly/Bed 812 epoxide resin. The next day, the 1:1 mixture was replaced with 100% embedding medium at room temperature. The

membrane was detached using a small scapula, and a 5 microliter drop of 100% embedding medium was placed on a plastic Permanox slide (Thomas Scientific, Swedesboro, NJ) with the organoids facing upwards. A beem capsule filled with 100% embedding medium was then inverted on top of the membrane, with the attached organoids. The slides with the membrane/organoids and beem capsule was placed in a 60°C oven for 48 – 72 hours for polymerization. Semi-thin sections were cut and stained with 1% toluidine blue O in 1% sodium borate for examination with a light microscope. After trimming the block faces down to area of interest, ultrathin sections (90-100 nm thick) were cut from the selected blocks, placed on 100 mesh copper grids, and then stained with uranyl acetate and lead citrate. Digital images were captured with a Gatan Orius SC 1000 attached to an FEI Co. Tecnai T12 transmission electron microscope.

Reference:

Barkauskas CE, et al. Type 2 alveolar cells are stem cells in adult lung. *J Clin Invest.* 2013;123(7):3025-3036.

Normal lung (PBS)

A

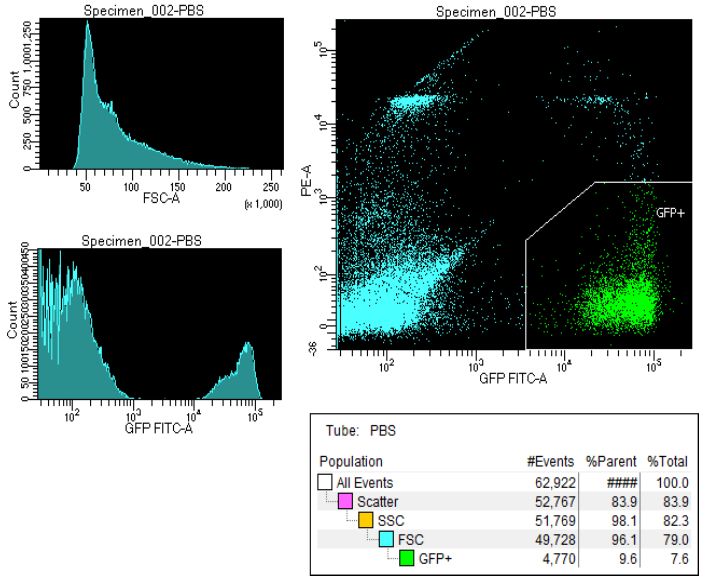

Tube: PBS

Population	#Events	%Parent	%Total
All Events	62,922	####	100.0
Scatter	52,767	83.9	83.9
SSC	51,769	98.1	82.3
FSC	49,728	96.1	79.0
GFP+	4,770	9.6	7.6

Fibrotic lung (Bleomycin)

B

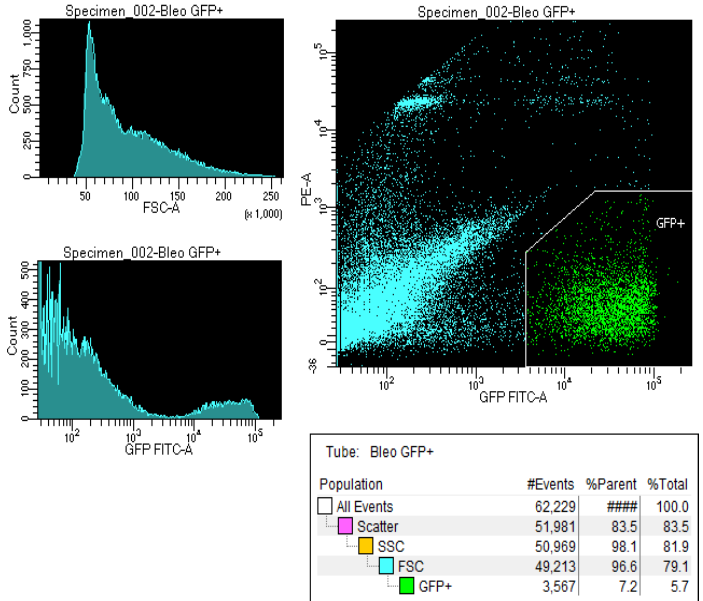

Tube: Bleo GFP+

Population	#Events	%Parent	%Total
All Events	62,229	####	100.0
Scatter	51,981	83.5	83.5
SSC	50,969	98.1	81.9
FSC	49,213	96.6	79.1
GFP+	3,567	7.2	5.7

C

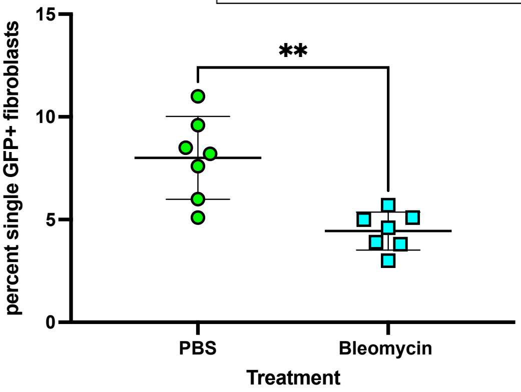

Supplemental Figure S1. Gating strategy for FACS isolation of GFP+ cells from *Pdgfrα-GFP* reporter mice dosed with either PBS (A) or 2 U/Kg bleomycin (B) for collection of live cells on day 14 post-exposure for scRNA-Seq. (C) Percent single GFP+ cells from FACS isolation of lungs from PBS or Bleomycin *Pdgfrα-GFP* mice over 7 independent experiments. *p*=0.0011. Statistics conducted with GraphPad Prism (v9.3.0) 2-tailed t-test.

A

B

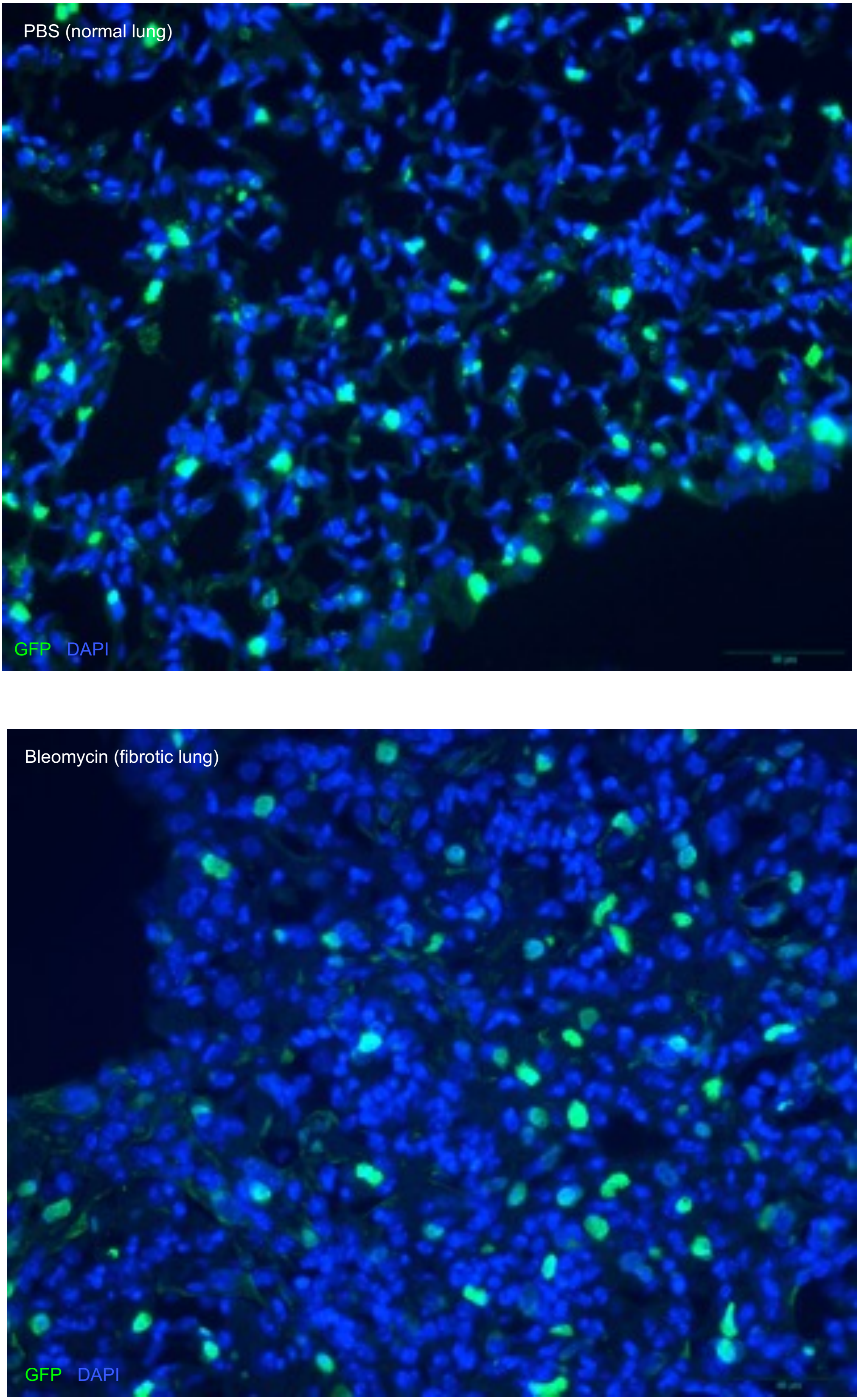

Supplemental Figure S2. Localization of GFP in (A) normal and (B) fibrotic *Pdgfrα-GFP* reporter mouse lungs.

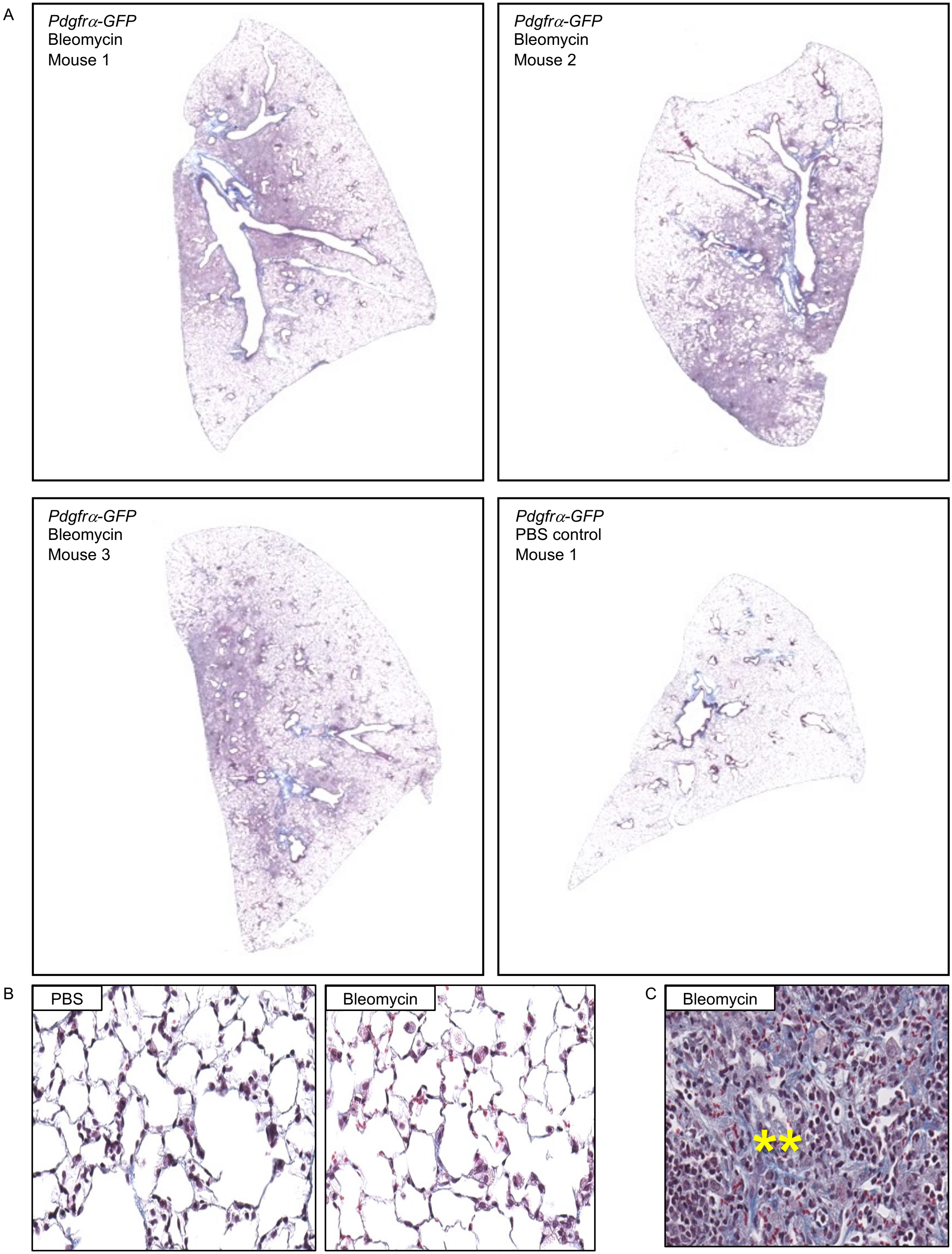

Supplemental Figure S3. Histological evaluation of fibrosis in *Pdgfrα-GFP* mice. (A) Left lung images are scaled to 1.2X. (B) 80X images of alveolar regions in PBS (top image) and unaffected regions in Bleomycin (lower panel) dosed mice.; (C) 80X image of Bleomycin injured area showing blue-stained collagen deposition (yellow asterisks).

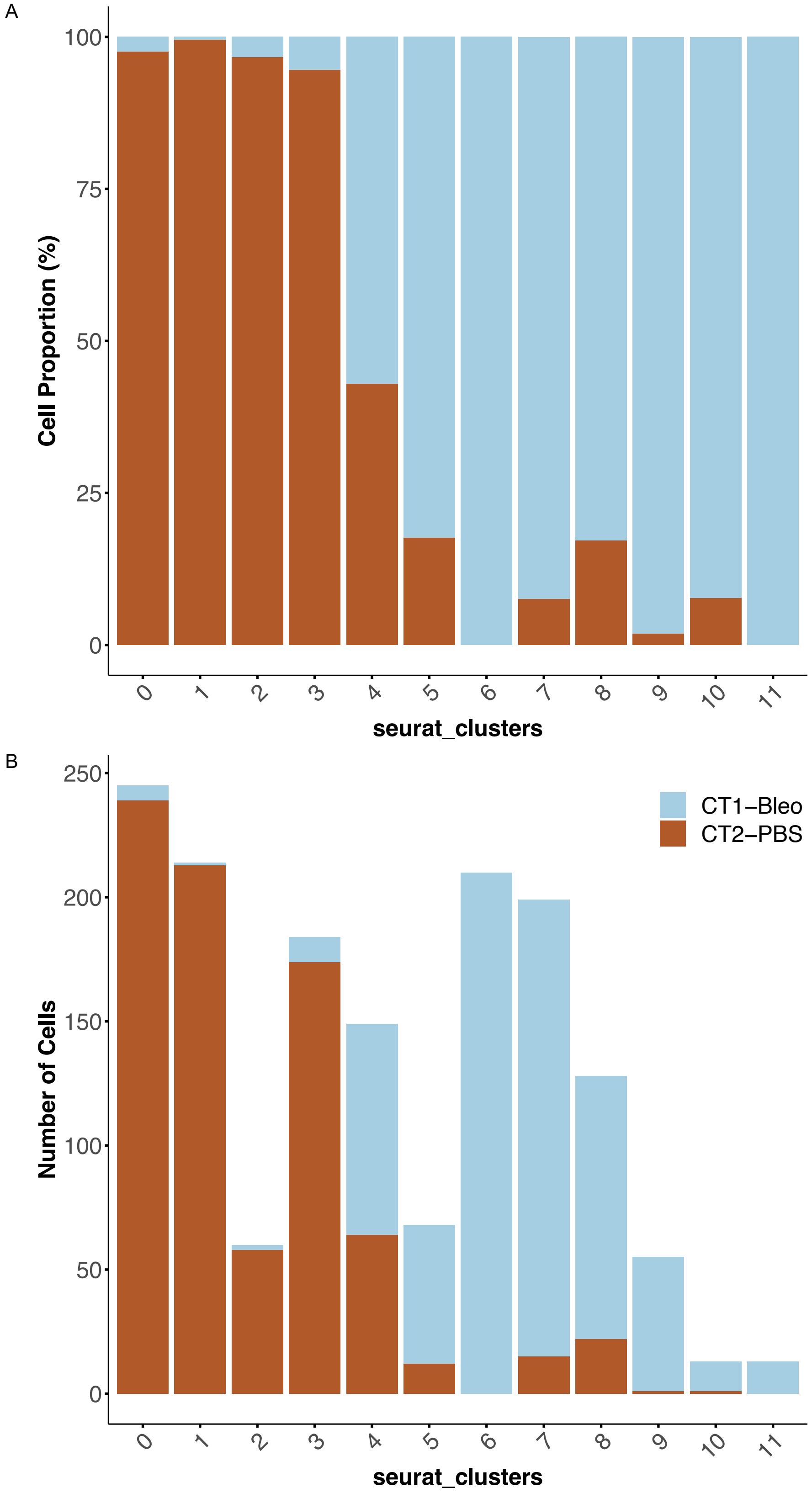

Supplemental Figure S4. (A) Cell proportion contribution and (B) number of cells from each treatment per cluster, comparing normal (PBS) and fibrotic (Bleomycin) *Pdgfraα-GFP* mice.

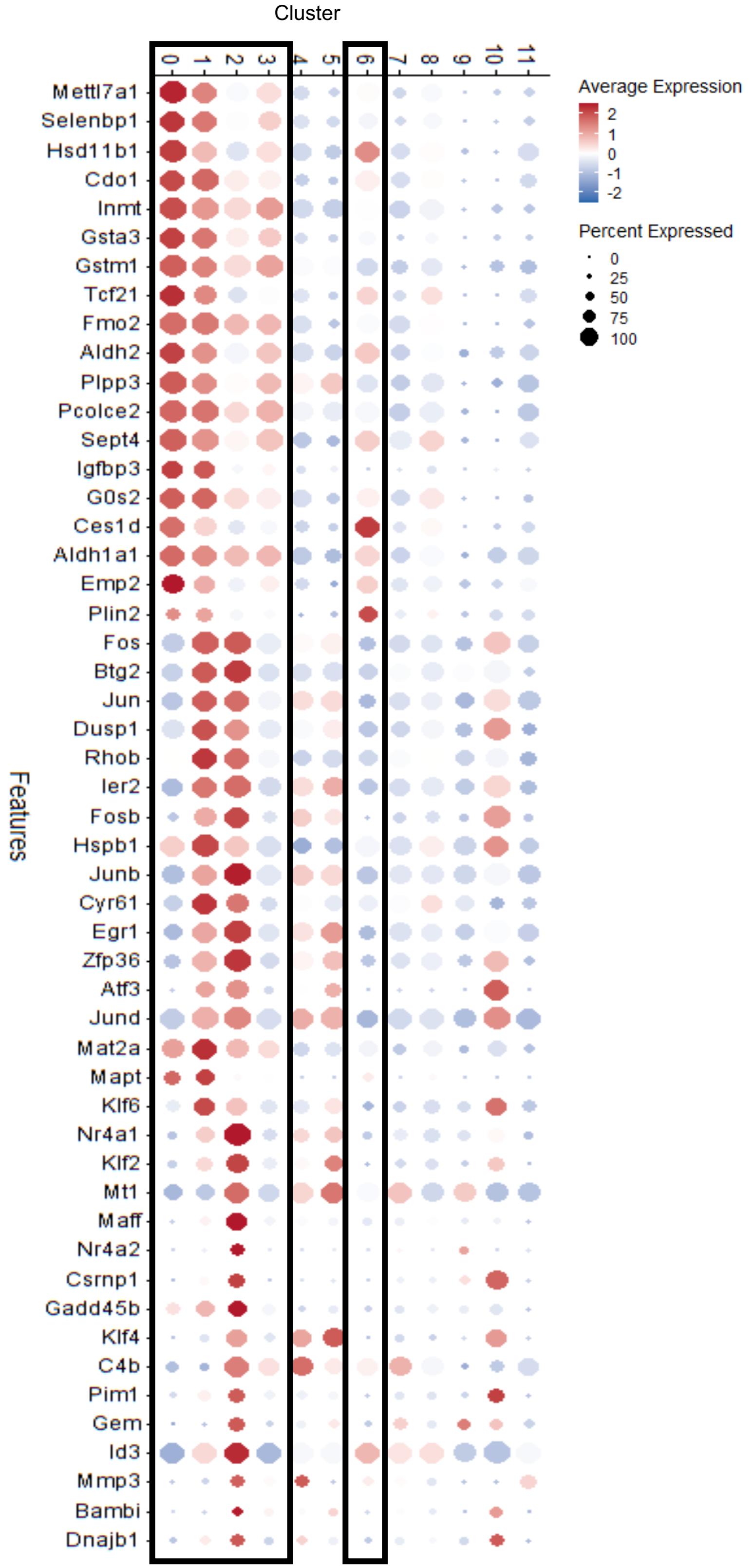

Supplemental Figure S5. Dot plot of genes characterizing lipofibroblast (LipoFB)-related clusters in PBS (clusters 0-3) and Bleomycin (clusters 6-8). Clusters 0 and 6 are the canonical LipoFB clusters for normal and fibrotic lung, respectively.

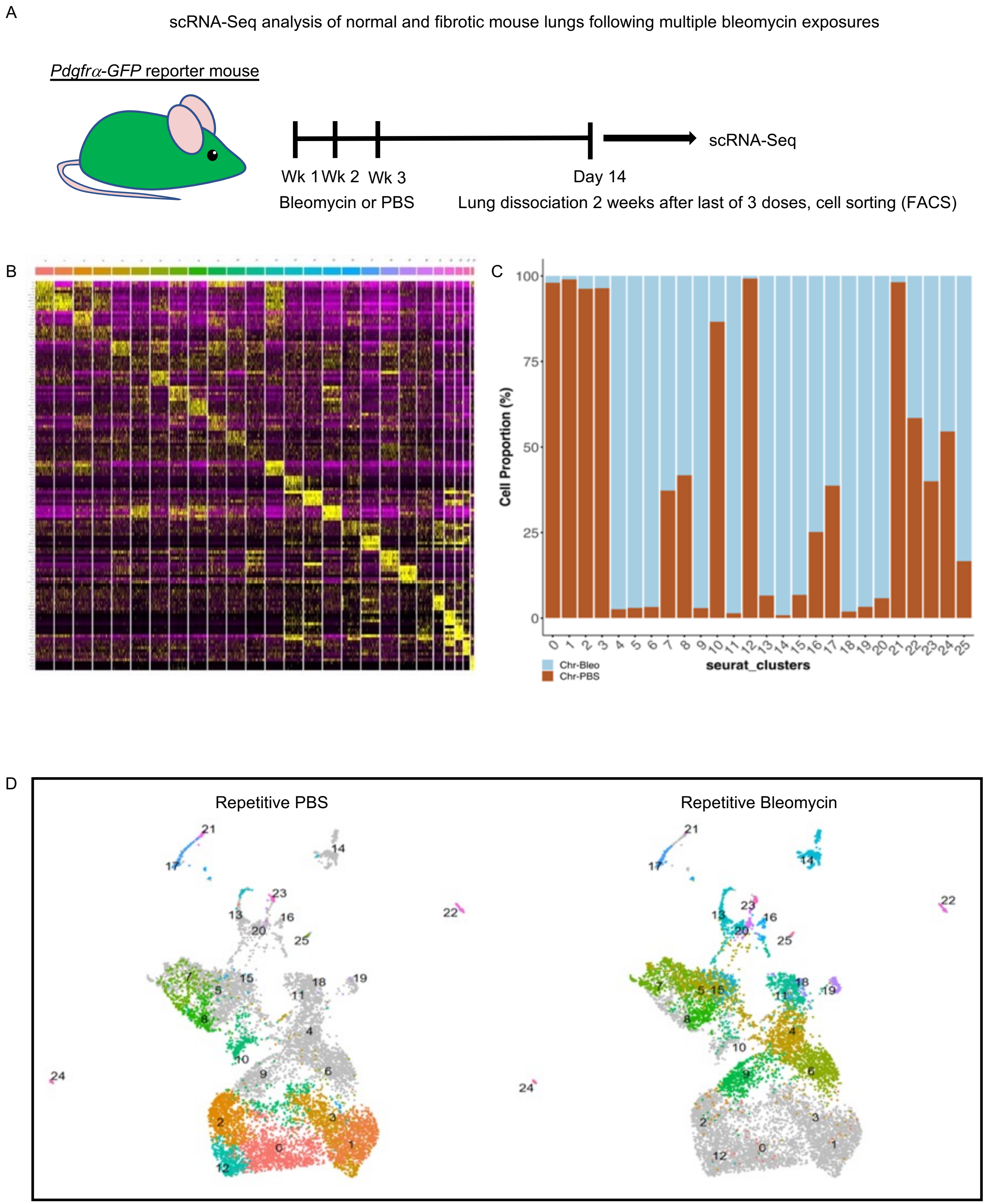

Supplemental Figure S6. scRNA-Seq analysis of repetitive bleomycin exposure. (A) Experimental model for repetitive bleomycin or PBS exposure of *Pdgfrα-GFP* reporter mice for scRNA-Seq analysis. (B) Heat map of joint analyzed individual clusters. (C) Proportion plot of clusters, Bleomycin versus PBS. (D) Split UMAP of PBS (left image) and Bleomycin (right image) to show treatment distribution across clusters.

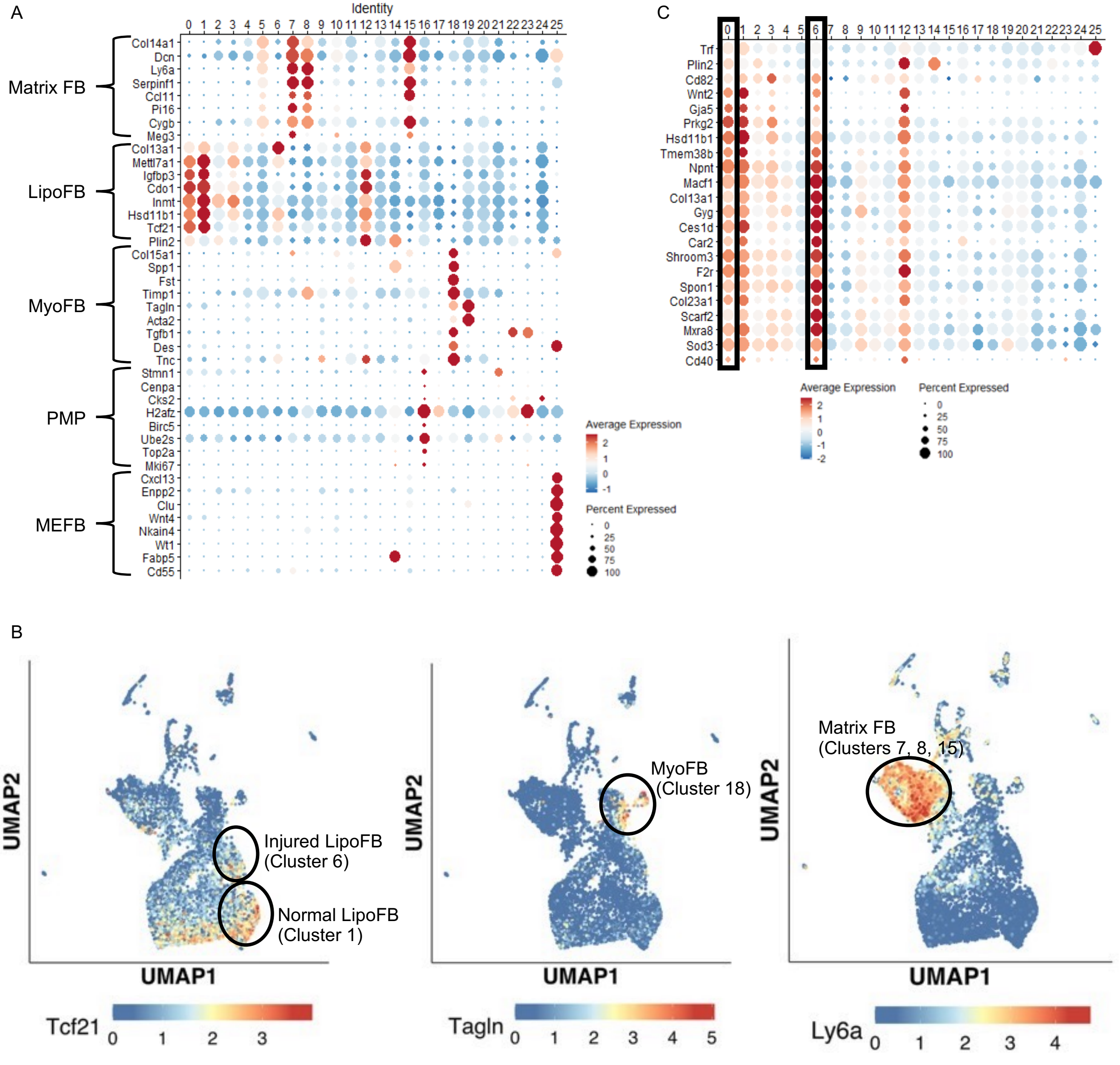

Supplemental Figure S7. scRNA-Seq analysis of repetitive bleomycin exposure. (A) Dot plot of cluster markers from Figure 1D using repetitive bleomycin/PBS scRNA-Seq data to identify individual cluster identities (Top to bottom: Matrix fibroblasts; LipoFB; MyoFB; PMP; MEFB). (B) Scatter plots of injured and normal LipoFB (top), MyoFB (middle) and Matrix FB (lower). (C) Dot plot using shared LipoFB signature genes from Figure 3 A and B with scRNA-Seq data from repetitive Bleomycin/PBS exposures, highlighting clusters 1 (normal) and 6 (injured LipoFBs), open boxes.

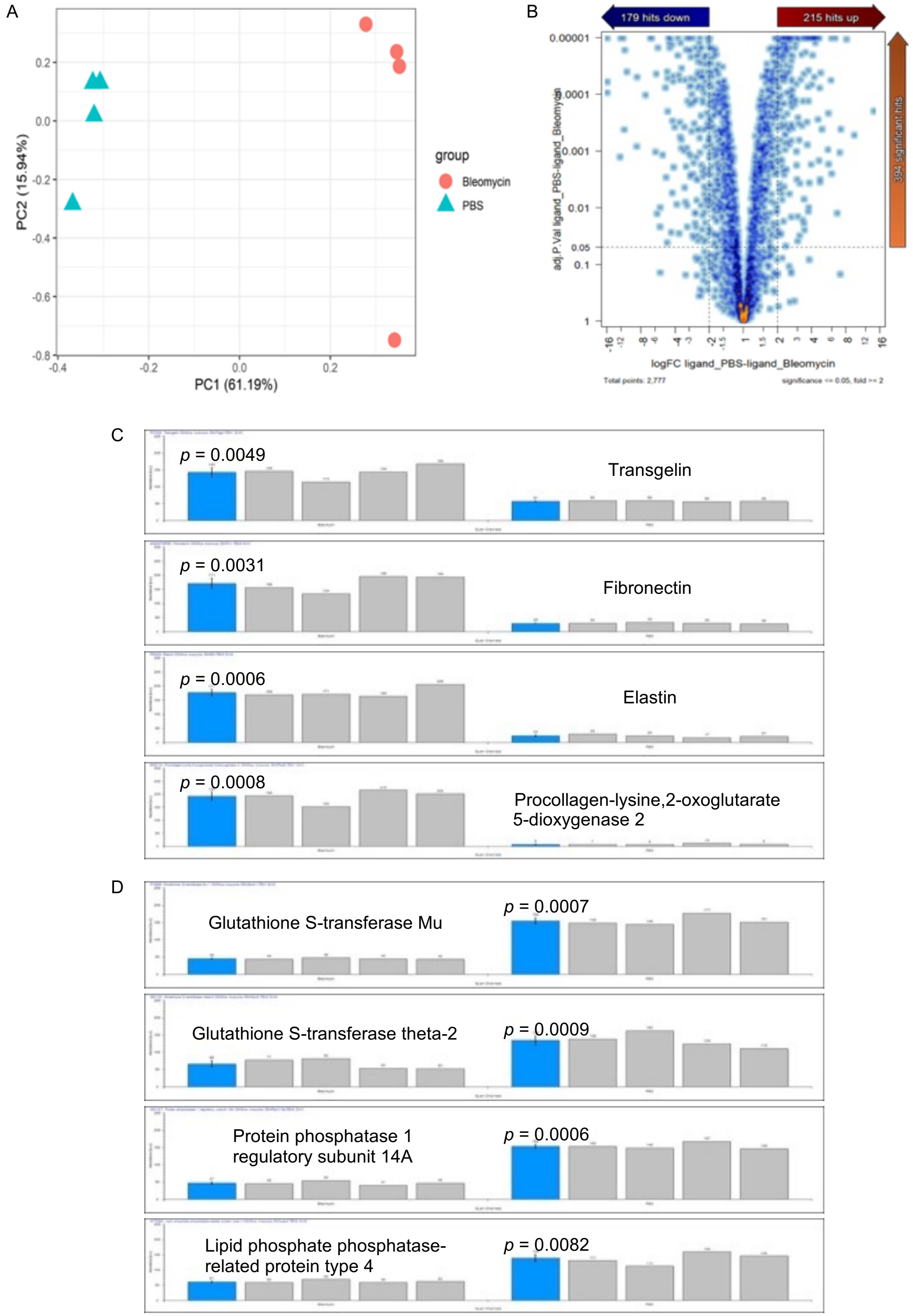

Supplemental Figure S8. Proteomics quality control. *Pdgfrα-GFP* mice were dosed with either Bleomycin or PBS as described in Figure 1A. Protein was isolated from a total of 4 biological replicates. (A) Principal Component Analysis (PCA) of PBS versus Bleomycin replicates. (B) Volcano Plot of differentially expressed protein; adjusted *p*-value <= 0.05 and fold change >= 2. (C) Bar charts of selected protein upregulated in Bleomycin; column in blue is average. (D) Bar charts of selected proteins upregulated in PBS. *p*-values were calculated in Proteome Discoverer, using 2-way ANOVA.

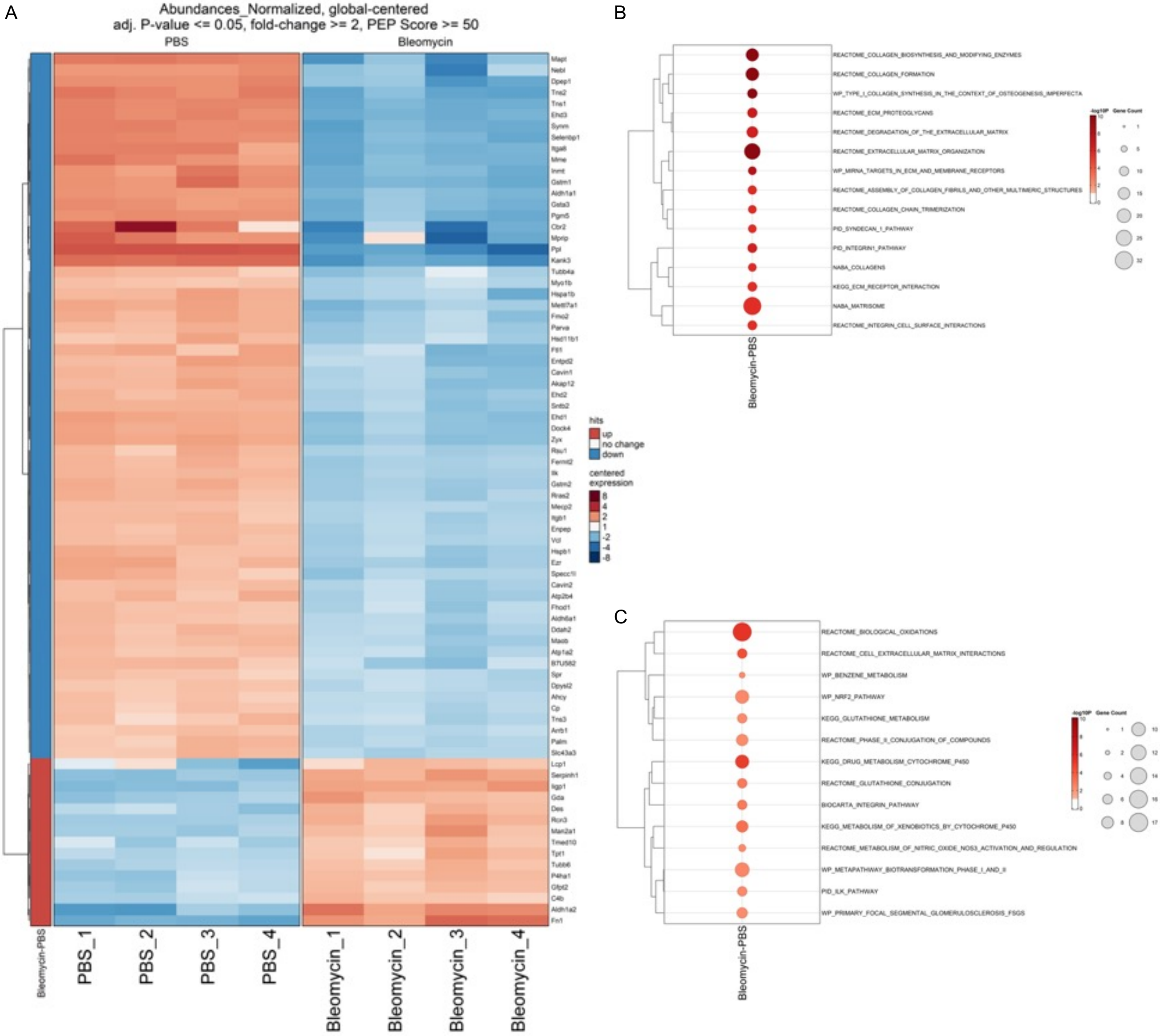

Supplemental Figure S9. (A) Heatmap comparison of up- and down-regulated proteins between PBS and Bleomyocin-dosed *Pdgfrα-GFP* reporter mice, filtered for *p*<=0.05, fold change =>2, and PEP Score >=50. The left-hand panel shows PBS/Bleomycin expression change. (B) Pathways enriched (-Log10P>=1) identified by Gene set enrichment analysis (GSEA) of proteins enriched (*p*<=0.05, fold change =>2) in Bleomycin-dosed samples. Gene set generated from Human Molecular Signatures Database (MSigDB) Canonical pathways. (C) GSEA analysis of proteins down-regulated in Bleomycin-dosed samples.

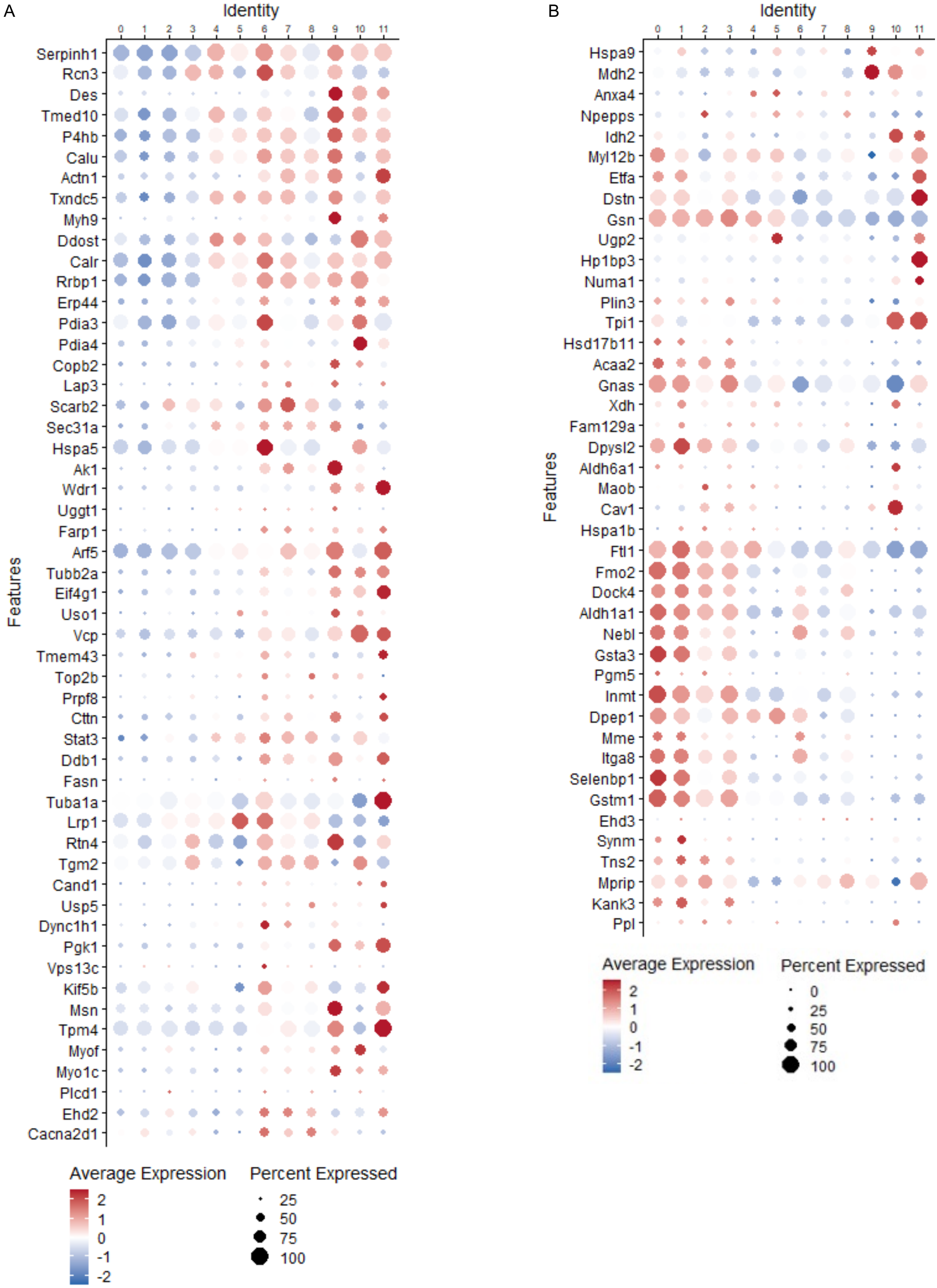

Supplemental Figure S10. (A) Dot plot of proteins identified as upregulated in injured LipoFB, downregulated in normal. (B) Dot plot of proteins identified as downregulated in injured LipoFB, up in normal LipoF. Dot plots were generated using scRNA-Seq cluster markers from Supplemental Table 2.

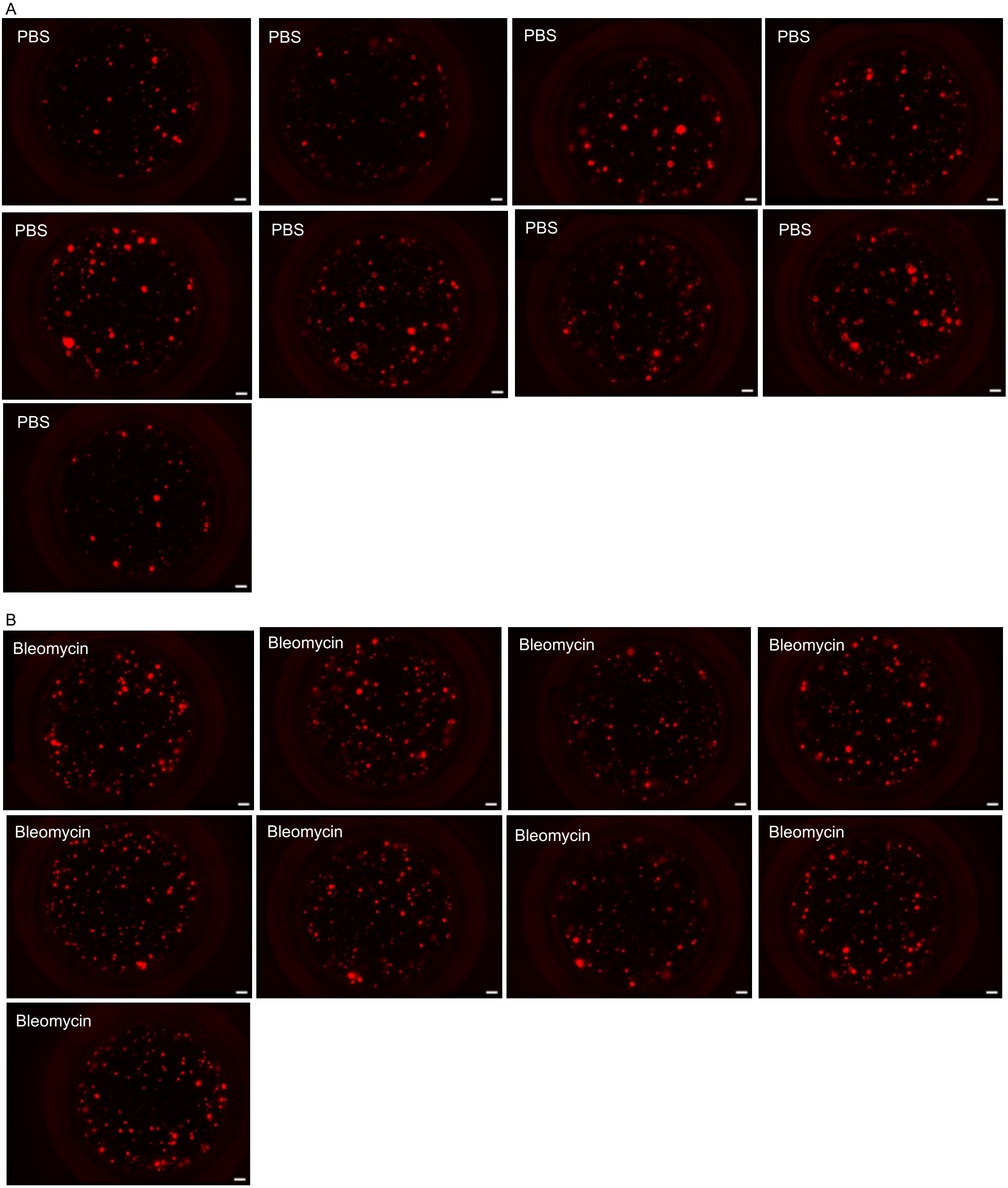

Supplemental Figure S11. Epifluorescence images of alveolosphere growth in individual wells on day 14. Cultures were co-cultured with SFTPC-tomato+ AEC2 cells and GFP+ fibroblasts from (A) PBS or (B) Bleomycin-exposed *Pdgfrα-GFP* reporter mice. Images were captured using an automated Epifluorescence microscope (Zeiss), scale bars equal 500 microns.

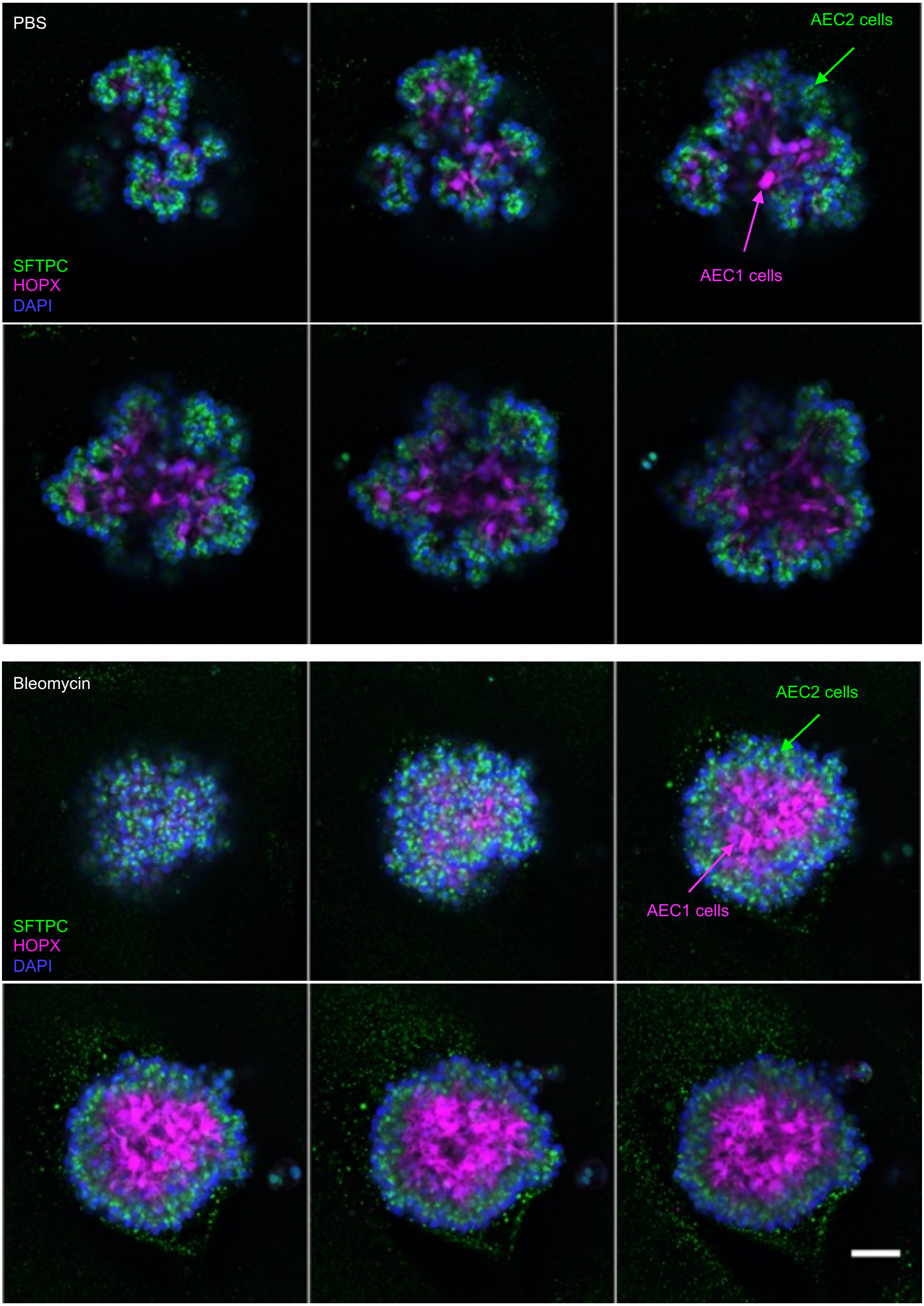

Supplemental Figure S12. Alveolospheres grown with fibrotic *Pdgfrα*+ FB exhibit a condensed morphology. Series of Z-stacks (n=6) from confocal images taken of whole mount stained alveolospheres (PBS top panels, Bleomycin lower panels) showing differences in colony morphology. Scale bar = 50 microns.

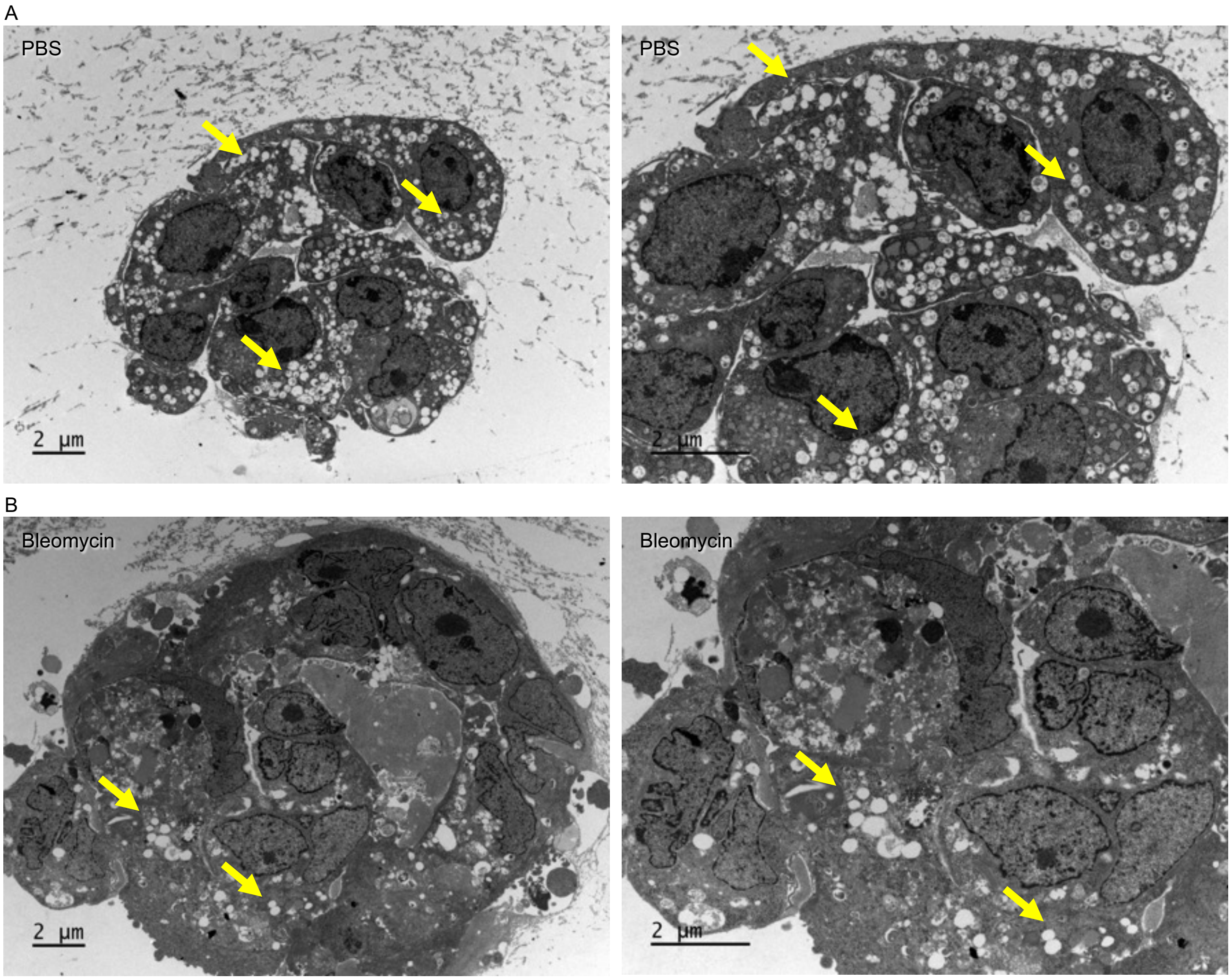

Supplemental Figure S13. Transmission Electron Microscopy (TEM) micrographs of alveolospheres grown from (A) PBS-exposed (normal) *Pdgfrα+* fibroblasts, top two images, and (B) Bleomycin-exposed (fibrogenic) fibroblasts, lower two images. Arrows indicate lipid-filled vesicles. Scale bars are 2 microns.

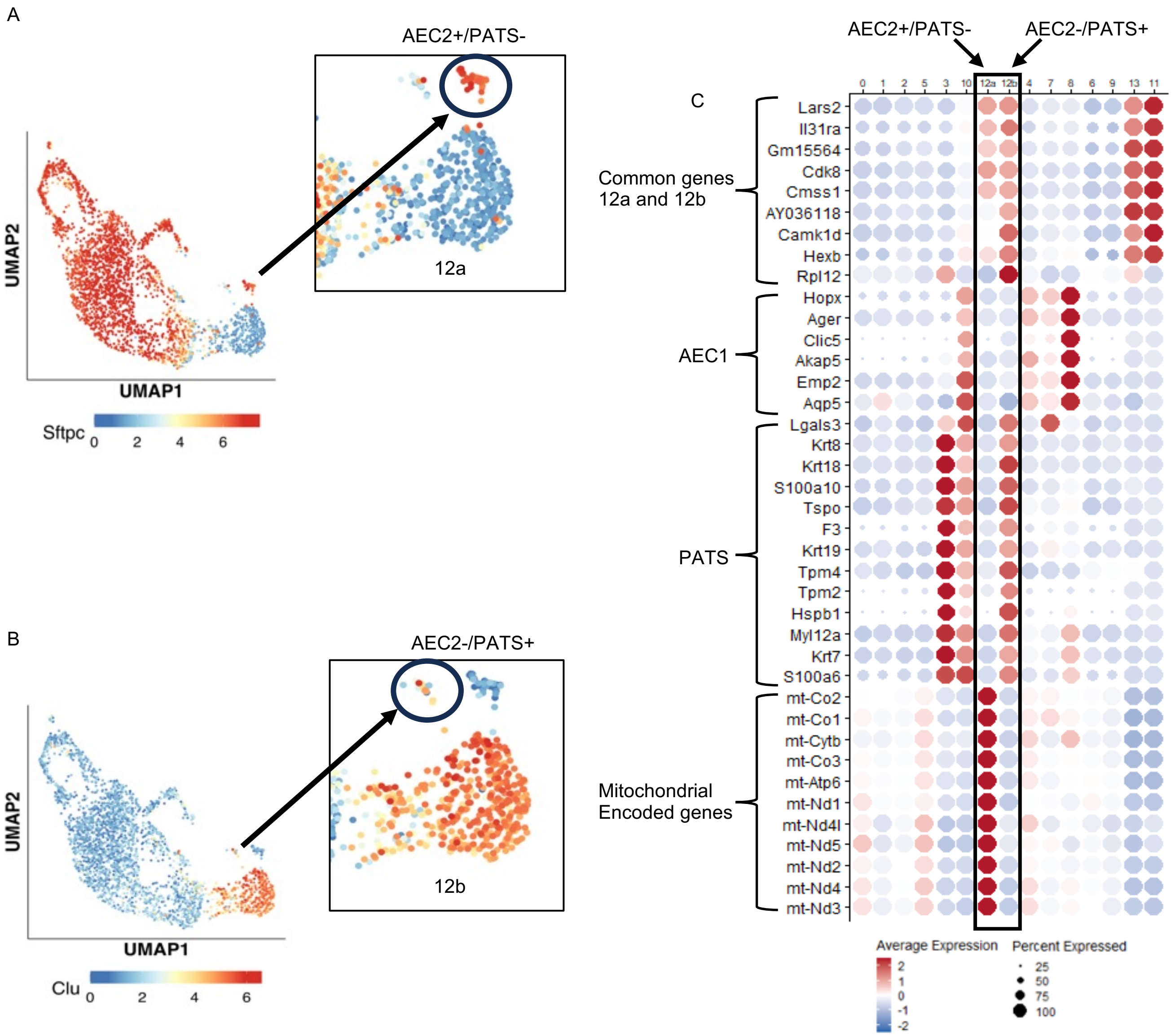

Supplemental Figure S14. (A) Scatter plot of AEC2 marker *Sftpc*, inset showing expression in cluster 12 subset a. (B) Scatter plot of the PATS marker Clusterin (*Clu*), inset showing expression in cluster 12, subset b. (C) Dot plot of common genes for cluster 12 subsets a and b; AEC1 markers; PATS markers; and mitochondrial encoded genes.

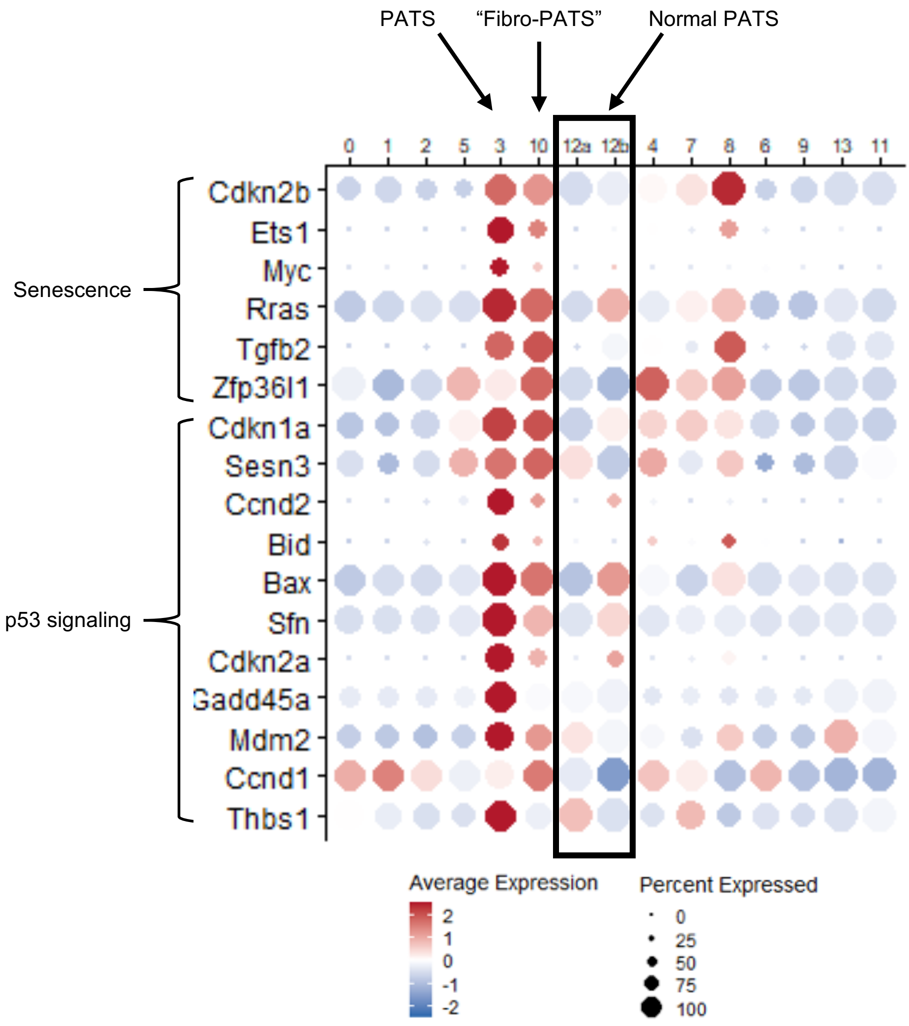

Supplemental Figure S15. Dot plot of senescence and p53 signaling markers.

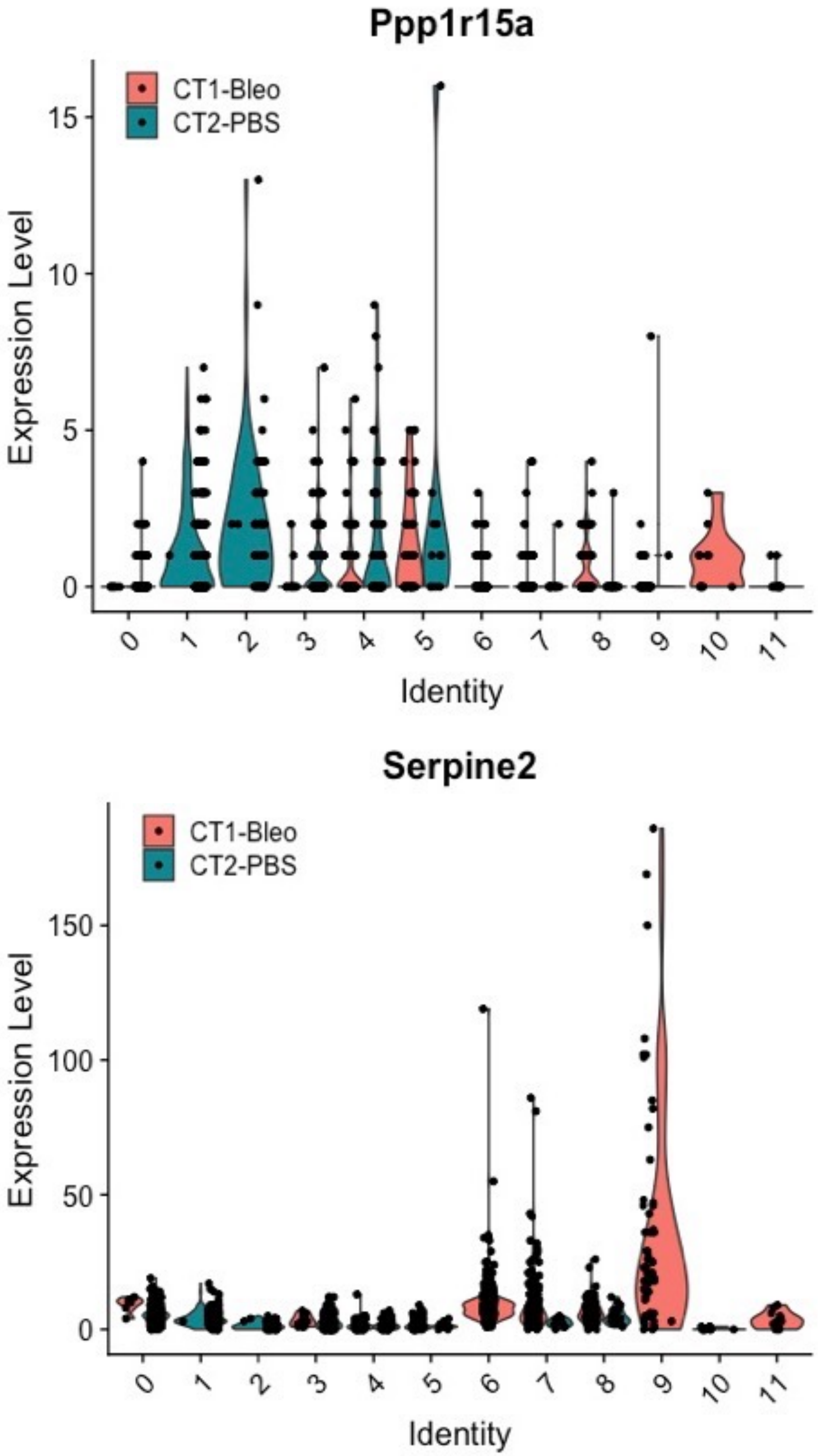

Supplemental Figure S16. Split violin plots showing senescence markers *Pp1r15a* and *Serpine2* expression levels across clusters.
